# A NIMA-Related Kinase Suppresses the Flagellar Instability Associated with the Loss of Multiple Axonemal Structures

**DOI:** 10.1371/journal.pgen.1005508

**Published:** 2015-09-08

**Authors:** Huawen Lin, Zhengyan Zhang, Suyang Guo, Fan Chen, Jonathan M. Kessler, Yan Mei Wang, Susan K. Dutcher

**Affiliations:** 1 Department of Genetics, Washington University School of Medicine, St. Louis, Missouri, United States of America; 2 Department of Physics, Washington University in St. Louis, St. Louis, Missouri, United States of America; Massachusetts General Hospital, UNITED STATES

## Abstract

CCDC39 and CCDC40 were first identified as causative mutations in primary ciliary dyskinesia patients; cilia from patients show disorganized microtubules, and they are missing both N-DRC and inner dynein arms proteins. In *Chlamydomonas*, we used immunoblots and microtubule sliding assays to show that mutants in *CCDC40* (*PF7*) and *CCDC39* (*PF8*) fail to assemble N-DRC, several inner dynein arms, tektin, and CCDC39. Enrichment screens for suppression of *pf7*; *pf8* cells led to the isolation of five independent extragenic suppressors defined by four different mutations in a NIMA-related kinase, *CNK11*. These alleles partially rescue the flagellar length defect, but not the motility defect. The suppressor does not restore the missing N-DRC and inner dynein arm proteins. In addition, the *cnk11* mutations partially suppress the short flagella phenotype of N-DRC and axonemal dynein mutants, but do not suppress the motility defects. The *tpg1* mutation in *TTLL9*, a tubulin polyglutamylase, partially suppresses the length phenotype in the same axonemal dynein mutants. In contrast to *cnk11*, *tpg1* does not suppress the short flagella phenotype of *pf7*. The polyglutamylated tubulin in the proximal region that remains in the *tpg1* mutant is reduced further in the *pf7; tpg1* double mutant by immunofluorescence. CCDC40, which is needed for docking multiple other axonemal complexes, is needed for tubulin polyglutamylation in the proximal end of the flagella. The CCDC39 and CCDC40 proteins are likely to be involved in recruiting another tubulin glutamylase(s) to the flagella. Another difference between *cnk11-1* and *tpg1* mutants is that *cnk11-1* cells show a faster turnover rate of tubulin at the flagellar tip than in wild-type flagella and *tpg1* flagella show a slower rate. The double mutant shows a turnover rate similar to *tpg1*, which suggests the faster turnover rate in *cnk11-1* flagella requires polyglutamylation. Thus, we hypothesize that many short flagella mutants in *Chlamydomonas* have increased instability of axonemal microtubules. Both CNK11 and tubulin polyglutamylation play roles in regulating the stability of axonemal microtubules.

## Author Summary

Cilia are specialized projections found on the surface of eukaryotic cells. They play crucial sensory functions, as well as motile functions needed for clearing airways or propelling cells. Ciliary motility is perturbed in the inherited disease, Primary Ciliary Dyskinesia (PCD). Two coiled coil domain-containing (CCDC39 and CCDC40) proteins are needed for the assembly of multiple key structures/complexes that are required for generating ciliary motility. Using the unicellular green alga, *Chlamydomonas*, we have identified a kinase (CNK11) that when mutated is able to partially rescue the short flagella phenotype of the *ccdc39* and *ccdc40* mutants as well as mutants lacking axonemal dyneins or the N-DRC complex. In addition, CCDC40 is required for tubulin polyglutamylation at the proximal end of flagella. We suggest that substructures like dynein arms and the N-DRC, which are needed for motility, play a second role in stabilizing the axonemal microtubules and are needed for proper length control. The polyglutamylase, TTLL9, and the kinase, CNK11, play roles in stabilizing the axonemal microtubules based on their ability to partially rescue the short flagella phenotypes of multiple mutants.

## Introduction

Defects in ciliary assembly and function cause a wide range of human diseases and syndromes called ciliopathies. Primary ciliary dyskinesia (PCD) is diagnosed by defects in ciliary motility, and is associated with a genetically heterogeneous group of recessive disorders [1]. Mutations causing PCD have been identified in genes encoding axonemal dynein subunits [2, 3], dynein assembly factors [4–6], and dynein docking/adaptor factors [7, 8]. The nexin-dynein regulatory complex (N-DRC) is an axonemal structure critical for the regulation of dynein motors and for connecting doublet microtubules to each other. Loss-of-function mutations in *DRC1* (*CCDC164/PF3*) and *DRC3* (*CCDC65*) cause severe defects in assembly of the N-DRC structure and result in defective ciliary movement in humans and *Chlamydomonas reinhardtii* [6, 9, 10]. *PF2*, which encodes DRC4, was used to identify 11 proteins in the N-DRC [10]. Mutations in *CCDC39* and *CCDC40* cause altered ciliary beating with the disorganization of the axoneme that includes the displacement of the peripheral outer doublets, as well as central pair microtubules, radial spokes and inner dynein arm defects [11–15]. Loss-of-function mutations in *CCDC39* and *CCDC40* in *Chlamydomonas* lead to short flagella, irregularly spaced radial spokes, absence or reduction of N-DRC components and inner dynein arm proteins [16, 17]. *CCDC39* and *CCDC40* mutations in children lead to earlier and more severe lung disease than in PCD patients with outer dynein arm mutations [18].

In *Chlamydomonas*, there are many mutations that can lead to short flagella. Partial reduction in IFT proteins (IFT144 (*FLA15*) and IFT139 (*FLA17*)) or motors such as the kinesin-2 motor FLA10 or cytoplasmic dynein result in short flagella [19–21]. Changes in the cytoplasmic pool of tubulins and flagellar precursor proteins also affect flagellar length [22, 23]. In addition, the simultaneous loss of multiple substructures, such as the dynein arms, radial spokes, and the N-DRC, result in short flagella [24–26]. LeDizet and Piperno isolated a suppressor (*ssh1*) that increased the flagellar length in double mutant strains that lacked outer and inner dynein arms without restoring the missing structures [26]. A recent study identified a deletion in the *TPG2* gene as the causative mutation in the *ssh1* strain [27]. *TPG2* encodes FAP234, a flagellar protein that forms a complex with a tubulin polyglutamylase TTLL9/TPG1 [28, 29]. Tubulin polyglutamylation adds multiple glutamates to both α- and β-tubulin subunits along microtubules in cilia/flagella, basal bodies, and neuron axons [30–32]. Several tubulin tyrosine ligase-like (TTLL) proteins carry out the polyglutamylation process. Tubulin polyglutamylation can affect microtubule assembly, stability, and motility [32]. In *Chlamydomonas*, *tpg1* affects polyglutamylation of α-tubulin specifically and shows a flagellar motility defect [29]. Both *tpg1* and *tpg2* mutations suppress the short flagella phenotype found in mutants that lack multiple axonemal dynein species [27].

NIMA-related protein kinases have been found in eukaryotes and their functions are related to regulation of cell cycle, cilia length, and microtubule stability [33–38]. Currently, there are 11 NIMA-related protein kinases identified in *Chlamydomonas* [33] and only two of them have been functionally characterized [35, 36]. A null mutant of the NIMA-like protein kinase *CNK2* in *Chlamydomonas* has slightly longer flagella and defective flagellar disassembly. The *cnk2-1* mutant has decreased tubulin turnover at the flagellar tip, which suggests that a reduced rate of flagellar disassembly is compensated by reduced rate of assembly [36]. The CNK2 protein, together with a MAP kinase (LF4), respond to flagellar length signals and block assembly and promote disassembly, respectively [36]. Thus, they provide input to the balance between assembly and disassembly of axonemal microtubules and flagellar length.

In this study, we identify a novel NIMA-related protein kinase CNK11 that rescues the short flagella phenotype found in several N-DRC mutants, as well as mutants lacking dynein arms. In addition, we discovered that the polyglutamylation defect caused by *tpg1* could not rescue the *CCDC40* mutant. Instead, the *CCDC40* mutation in the *tpg1* background has narrower distribution of polyglutamylated tubulin at the proximal end of flagella. The microtubule stabilizing drug paclitaxel is able to rescue the short flagella phenotype in *CCDC39*/*CCDC40* mutants but this rescue fails in the presence of *cnk11* or *tpg1*.

## Results

### Identification of the causative mutations in *pf7*, *pf8* and *fla12*

**Fig 1:**
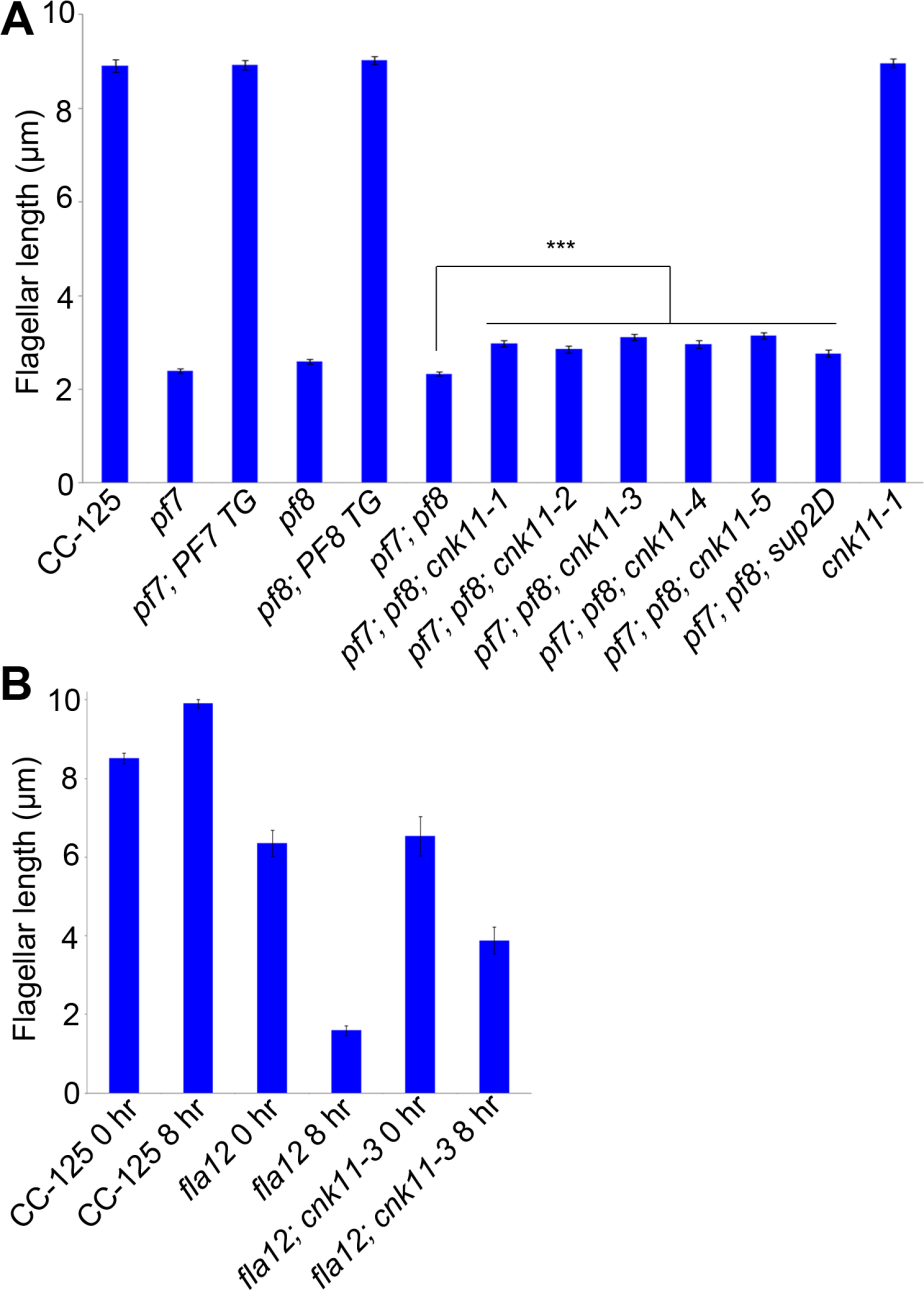
Flagellar length of *ccdc39/ccdc40* mutants can be partially rescued by the *cnk11* suppressors. At least 100 flagella from each strain were measured to determine the average flagellar length. Error bars represent standard deviation of the mean. (A) Flagellar length of various strains at 21°C. TG, transgene. *** indicates p<0.001 by t-test. (B) Flagellar length of various strains before (0 hr) and after (8 hr) temperature shift to 32°C.

The *pf7* and *pf8* mutants were first isolated as mutants with no flagella or short flagella with a motility defect [17, 39] (Fig 1), and mapped to chromosome 17 [40, 41]. Using whole genome sequencing in combination with our SNP and short insertion/deletion library, we identified the causative mutations in both *pf7* and *pf8* mutant strains [42] (Table 1). A nonsense mutation in *Cre17*.*g698365* (*CCDC40*) is responsible for the *pf7* mutant phenotype; a nonsense mutation in *Cre17*.*g701250* (*CCDC39*) leads to the *pf8* mutation (Table 2). We performed BAC rescue to confirm they are the causative mutations (Fig 1A). Forty-one independent transformants that contain BAC DNA 17F4, which carries the *CCDC40* gene, showed restoration of both flagellar length and motility in *pf7*; 20 independent transformants that contain BAC DNA 31N18, which carries the *CCDC39* gene, restored flagellar length and motility in *pf8*. For each rescue, we analyzed 16 independent transformants and the transformed BAC DNA cosegregates with rescue in all transformants. Independently, Oda *et al*. showed that *pf7* and *pf8* encode CCDC40 and CCDC39 [16].

**Table 1:** Whole genome sequencing of mutant strains used in this study.

Strains	Index	Total reads	Aligned reads	Cover-age	% Aligned	# Total Changes	# Unique changes	# Putative changes*	# Changes within mapping intervals	Gene
*pf7*	TGAGGTT	113958070	106694994	90	94	67691	1505	4	1	*CCDC40*
*pf8*	GCTTAGA	104812850	97145742	82	93	73576	1423	4	2	*CCDC39*
*fla12*	TGAGGTT	51316544	31759955	27	62	51987	27145	43	2	*CCDC39*
*pf7; pf8*, *cnk11-1*	AATTCAT	49851384	37785929	32	76	104759	41459	9	n/a	*CNK11*
*pf7; pf8; cnk11-2*	TTTGGCG	50434300	39846537	34	79	74763	25267	8	n/a	*CNK11*
*pf7; pf8; cnk11-3*	AGGCGAA	52468650	47265275	40	90	81264	10272	59	n/a	*CNK11*
*pf7; pf8; cnk11-4*	CCGATTA	63095732	60194808	51	95	49140	1505	18	n/a	*CNK11*
*pf7; pf8; cnk11-5*	TAACAAG	49771244	38235224	32	77	90782	35558	11	n/a	*CNK11*

* Changes are defined as being within coding regions or at intron-exon boundaries and have a Phred quality score ≥100.

**Table 2:** Sequence changes found in individual mutant strains.

Strain	Chromosome	Position	Gene ID	Number of reads	Nucleotide change	Coding region change	Annotation
***pf7***	17	334588	Cre17.g698365	85	G to A	Q to stop	CCDC40
***pf8***	17	739540	Cre17.g701250	77	C to T	Q to stop	CCDC39
	17	285493	Cre17.g698233	30	Insertion of GCC	Insertion of G	Uncharacterized protein
***fla12***	17	746129	Cre17.g701250	14	T to C	L to P	CCDC39
	17	5735025	Cre17.g739050	14	G to A	G to D	Uncharacterized protein
***cnk11-1***	7	3893009–3893010	Cre07.g339100	15	CC to AG	S to stop	NIMA-like protein kinase
***cnk11-2***	7	3895322–3895323	Cre07.g339100	50	CT to TA	L to stop	NIMA-like protein kinase
***cnk11-3***	7	3899708	Cre07.g339100	74	ΔG	Frame shift	NIMA-like protein kinase
***cnk11-4***	7	3895368	Cre07.g339100	46	C to T	A to V	NIMA-like protein kinase
***cnk11-5***	7	3899708	Cre07.g339100	4	ΔG	Frame shift	NIMA-like protein kinase

The *fla12* mutant was isolated as a temperature-sensitive flagellar assembly mutant [43] that was previously mapped to chromosome 17 [40]. The *fla12* cells shorten their flagella gradually and become immotile after the temperature is raised from 21°C to 32°C (Fig 1B). We used the same whole genome sequencing approach to identify a L_845_P change in *CCDC39* in *fla12* (Tables 1 and 2). The transgene that rescued the *pf8* mutant was introduced into *fla12* by meiotic crosses. In 12 independent progeny, the transgene restores normal flagellar length and motility in all strains at 32°C.

### Suppressor/revertant screen of *pf7*, *pf8*, *fla12* and *pf7; pf8*

*Chlamydomonas* offers the ability to use suppressor analysis to find genes that restore function to motility mutants [44, 45]. After UV mutagenesis of the *pf7* mutant, we screened for swimming cells and recovered 31 independent strains. PCR/enzyme digestion and Sanger sequencing revealed that all 31 strains are intragenic revertants (Table 3). Using the same strategy, we isolated 34 revertants of *pf8* and 4 revertants of *fla12* (Table 3), all are intragenic events.

**Table 3:** Reversion of *pf7*, *pf8*, and *fla12* mutations.

**Intragenic revertants in *pf7* with TAG nonsense mutation**						
Wild-type (CAG)	TAG to CAG	TAG to TTG	TAG to TGG	TAG to GAG	TAG to AAG	
Glutamine	Glutamine	Leucine	Tryptophan	Glutamic acid	Lysine	
	23	3	1	2	2	
**Intragenic revertants in *pf8* with TAA nonsense mutation**							
Wild-type (CAA)	TAA to CAA	TAA to TCA	TAA to TAC	TAA to TTA	TAA to AAA	TAA to GAA
Glutamine	Glutamine	Serine	Tyrosine	Leucine	Lysine	Glutamic acid
	19	9	2	2	1	1
**Intragenic revertants in *fla12* with CCG (proline) missense mutation**							
Wild-type (CTG)	CCG to CTG	CCG to TCG	CCG to CAG	CCG to TTG		
Leucine	Leucine	Serine	Glutamine	Leucine		
	1	1	1	1		

Subsequently, we performed two independent UV mutagenesis screens on *pf7*; *pf8* double mutants to isolate extragenic suppressors. In contrast to nitrogen-starved, autolysin-treated cells that assemble ~ 2 μm flagella (Fig 1A), the *pf7*; *pf8* mutant cells in nitrogen-containing medium are mostly aflagellate. The first UV mutagenesis screen led to isolation of three independent strains (*pf7*; *pf8*; *cnk11-1*, *pf7*; *pf8*; *cnk11-2*, and *pf7*; *pf8*; *cnk11-3*) and the second UV mutagenesis screen identified three additional strains (*pf7*; *pf8*; *cnk11-4*, *pf7*; *pf8*; *cnk11-5*, and *pf7*; *pf8*; *sup2D*). All six strains show partial suppression of the aflagellate phenotype of *pf7; pf8*, but do not suppress the motility defect (Fig 1A). None of them is linked to either *pf7* or *pf8*. They each contain one suppressor mutation based on crosses to the *pf7; pf8* parent; the aflagellate phenotype segregates 2:2. The suppressor mutations in five of the strains (*cnk11-1* to *cnk11-5*) are tightly linked to one another (S1 Table). Whole genome sequencing (Table 1) revealed that the five strains each carry a mutation in *Cre07*.*g339100*. The causative mutation in the sixth suppressor, *sup2D*, is currently under analysis.

**Fig 2:**
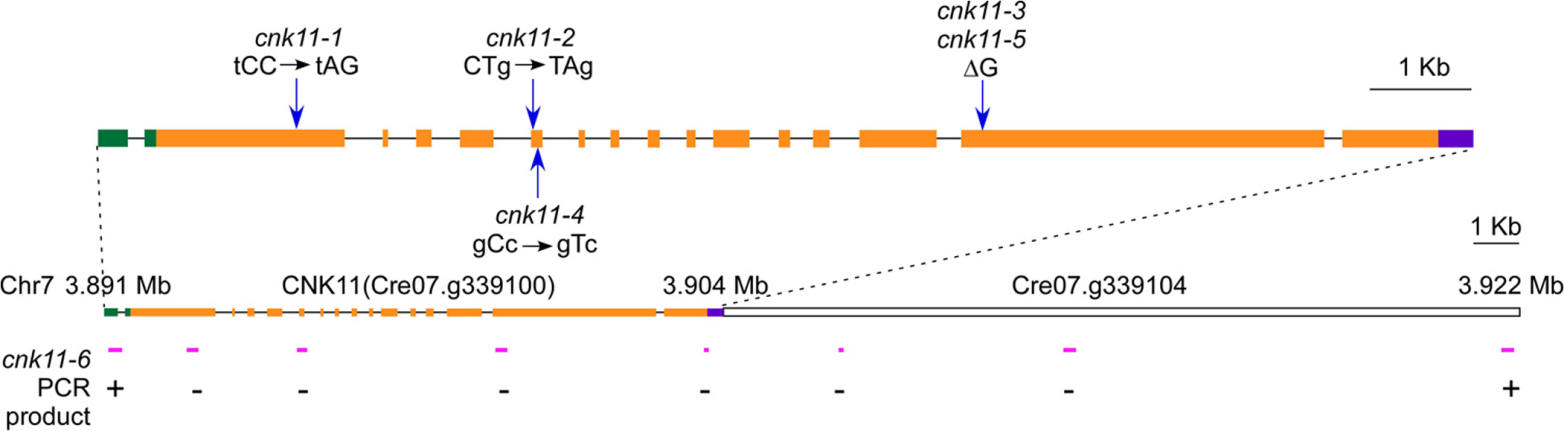
Gene structure of *CNK11* and its position on chromosome 7. Blue arrows indicate the relative positions of changes found in individual mutants. Green boxes, 5’ UTR; black solid lines, introns; orange boxes, exons; purple box, 3’ UTR. Magenta solid lines, relative positions of PCR products along *CNK11* and its neighboring gene *Cre07*.*g339104* in *cnk11-6*. +, PCR products amplified in both wild-type and *cnk11-6*;-, PCR products amplified in wild-type but not in *cnk11-6*.

In the five strains carrying mutations in *Cre07*.*g339100*, two nonsense mutations (*cnk11-1* and *cnk11-2*), a frame shift (*cnk11-3* and *cnk11-5*), and a missense mutation (*cnk11-4*, Table 2) were identified (Fig 2). Using dCAPs markers designed to each mutation (S2 Table), we observed linkage between suppression and the mutant allele in each strain. *Cre07*.*g339100* encodes a 2903 aa (amino acid) protein with a NIMA-like protein kinase (NEK) domain (aa 582–921). This protein is different from the 11 NEKs (CNK1—CNK10, and FA2) that have been previously annotated in *Chlamydomonas* [33]. Thus, we name it CNK11 (*C* *hlamydomonas* NIMA-like protein kinase 11). Using the conserved protein kinase domain, we constructed a phylogenetic tree with using the kinase domains found in 77 NEKs from *Arabidopsis*, *Aspergillus*, *C*. *elegans*, *Chlamydomonas*, *Dictyostelium*, *Drosophila*, human, mouse, *Trypanosoma*, rice, *Xenopus* and zebrafish (S1 Fig and S3 Table). The tree reveals that CNK11 is phylogenetically different from any of the known NEK classes.

### The *cnk11* suppressor fails to restore N-DRC proteins and other axonemal proteins in *pf7* and *pf8*

**Fig 3:**
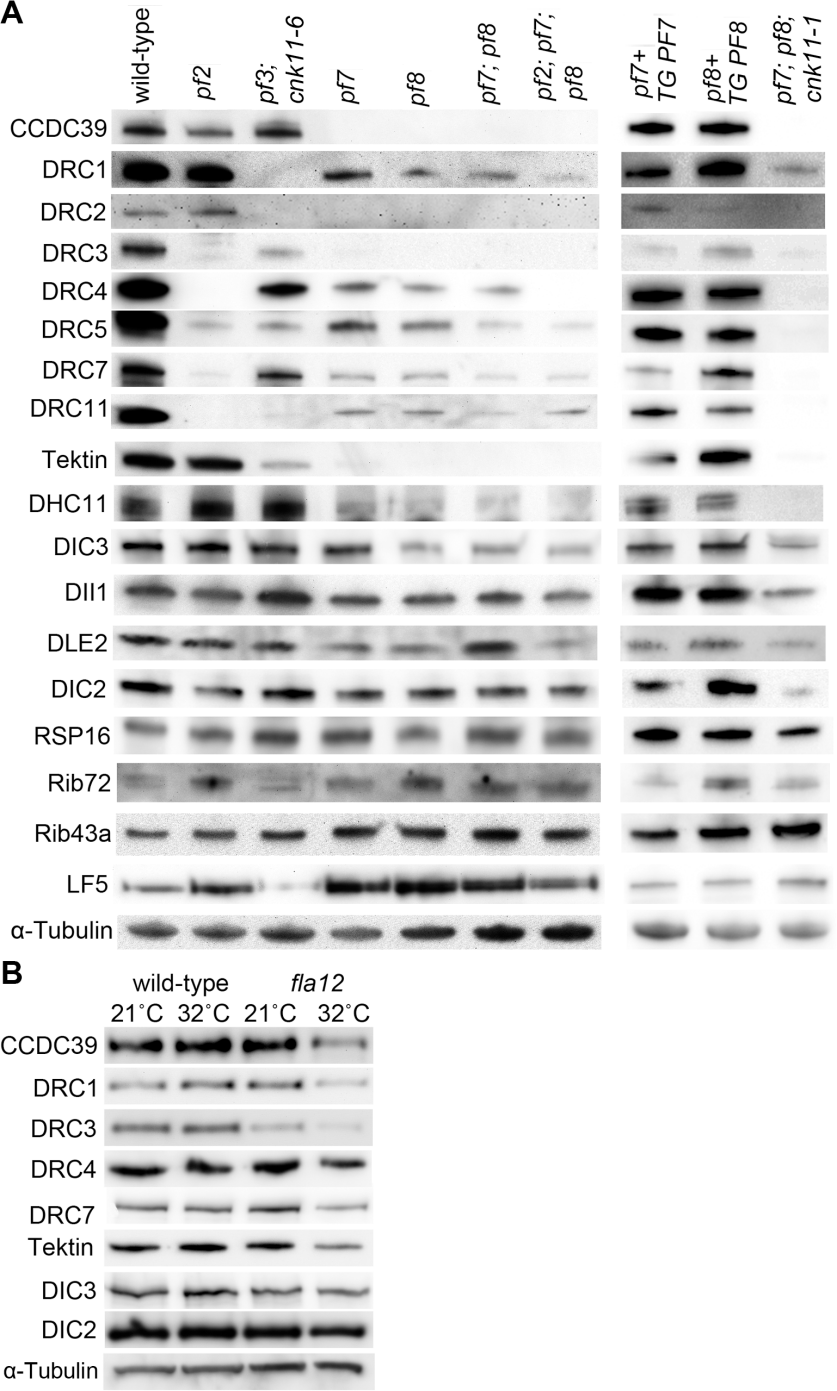
Reduced or absent N-DRC and other axonemal proteins are not restored in *pf7*; *pf8* suppressors. Ten micrograms of axonemes from various strains were used in the immunoblots. (A) Strains resuspended in nitrogen-free medium for 4 hours at 21°C. TG, transgene. (B) Cells were resuspended in nitrogen-free medium for 2 hours at 21°C. One-half of cells from each strain were switched to 32°C for 4 hours while the other half was maintained at 21°C for 4 hours. α-tubulin is included as a loading control.

In a previous report [16], Oda *et al*. showed that the *pf7* and *pf8* single mutants assemble reduced amounts of two N-DRC proteins; DRC2 and DRC4. In our analysis of isolated axonemes, we found that the single mutants assemble reduced amount of DRC1, DRC4, DRC5, DRC7, and DRC11 and completely lack DRC2, DRC3, and CCDC39 (Fig 3A). In addition, the amount of each N-DRC proteins in a *pf7; pf8* double mutant and a *pf2; pf7; pf8* triple mutant is comparable to that found in the single mutants, with the exception that no DRC4 protein is found in the triple mutant. This is expected given that the *pf2* mutant lacks DRC4 (Fig 3A) [10]. The similarity of the single and double mutants is also expected given the co-assembly of CCDC39 and CCDC40 [16]. Transformation of wild-type *PF7* or *PF8* into the corresponding mutant restores the N-DRC proteins (Fig 3A).

In axonemes from the *pf7; pf8; cnk11-1* mutant, the N-DRC proteins are not restored (Fig 3A). This suggests that *cnk11* mutants do not suppress the flagellar length defect of *pf7; pf8* via assembly of N-DRC proteins (Fig 3A). In addition to the N-DRC proteins, we asked if the *pf7* and *pf8* mutants affect other axonemal proteins (Fig 3A). Tektin, which is a microtubule binding protein, is diminished in the *ida6* (DRC1) and *pf3* (DRC2) mutants, which lack the inner dynein arms species *e* [46]. Tektin is missing in the single, double, and triple mutants (Fig 3A). Because loss of tektin is associated with the loss of dynein *e*, we suggest that these mutants are likely to lack dynein *e* as well. The proximally localized minor dynein heavy chain, DHC11 [47], is missing in the single, double, and triple mutants. DIC3/ IC140, which is the intermediate chain for the I1/*f* inner dynein arm [48, 49], is reduced in *pf8*, the double and triple mutants, but not in the *pf7* mutant. This is one of the few difference found between *pf7* and *pf8*, and was independently validated [16]. DLE2/centrin, which is part of the *b*, *e*, and *g* inner dynein arm complex [50, 51], is slightly reduced in the single mutants. There is no reduction of RSP16, which is one of the radial spoke proteins [52]; or DIC2/IC69, an intermediate chain in the outer dynein arm [53, 54]. DII1/p28 is only slightly reduced based on 2 independent preparations. Similar results were observed by Oda and colleagues [16]. The ribbon proteins, Rib72 and Rib43a, were first identified by their insolubility in various extraction protocols [55, 56]. There is no loss of these two proteins in the *pf7* or *pf8* mutant compared to wild-type or other N-DRC mutants. LF5, which is a CDKL5 homolog involved in length control that localizes to the proximal 1 μm of the flagella [57], is increased in the single, double, and triple mutants. Since we load equal amounts of protein in each sample, the increase is likely to be due to increased representation of proteins at the proximal end where LF5 localizes. The *pf7* and *pf8* rescued strains resemble wild-type axonemes and restore all proteins to wild-type levels. The triple mutant *pf7; pf8; cnk11-1* shows similar losses to the *pf7; pf8* preparations. It indicates that the *cnk11* mutant suppresses the *pf7; pf8* length phenotype by means other than restoration of axonemal proteins.

**Fig 4:**
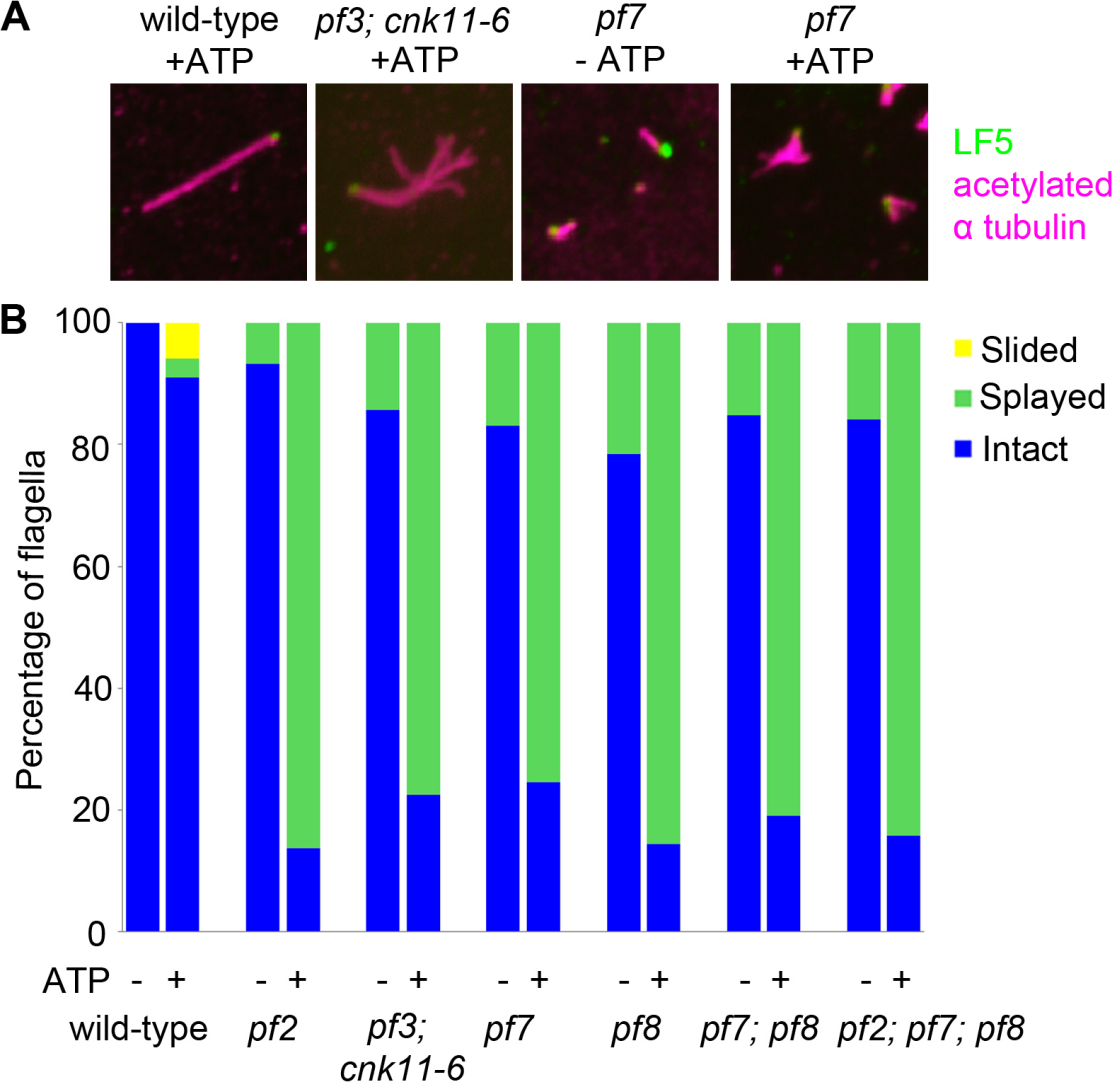
The *pf7* and *pf8* mutants show splaying phenotype in isolated axonemes similar to splaying in N-DRC mutants. (A) Fixed axonemes were stained with antibodies against LF5 (green) and acetylated α-tubulin (magenta). (B) Axonemes were scored as intact (blue), sliding (yellow), or splaying (green). At least 100 axonemes were score for each strain.

In N-DRC mutants such as *pf2* and *pf3*, the presence of 0.1 mM ATP leads to splaying of individual outer doublet microtubules in the medial and distal regions of the isolated full-length axonemes [10]. The proximal end of these axonemes remained intact. In contrast, wild-type axonemes remain intact throughout the whole length. Bower and colleagues concluded that the N-DRC provides some but not all of the resistance to microtubule sliding between doublets. This helps to maintain optimal alignment of doublets for productive flagellar motility [10]. Given *pf7* and *pf8* axonemes either lack or have reduced amounts of most N-DRC proteins tested, we examined isolated axonemes exposed to 0.1 mM ATP (Fig 4). To verify the proximal end showed splaying, we used antibodies to LF5 that localizes to the proximal end [57]. We observed little or no splaying of the doublet microtubules in wild-type axonemes. In the mutants, we observed splaying in the medial and distal regions of the single, double, and triple mutants that was similar to the splaying observed in N-DRC mutants [10]. The doublets in the proximal 1 μm end remain intact, similar to the observation found in N-DRC mutants. Thus, we conclude that CCDC39 and CCDC40 are not required for holding the microtubules together in the proximal region.

### The *fla12* mutant has normal IFT movement and its temperature-sensitivity is partially suppressed by *cnk11*

Given the temperature-sensitive *fla12* mutant carries a missense mutation in *CCDC39*, we asked whether the temperature shift affects axonemal proteins and flagellar length in this mutant. Four hours after temperature shift from 21°C to 32°C, *fla12* cells contain less CCDC39, DRC1, DRC3, DRC4, DRC7, and tektin while maintaining normal levels of DIC2 and DIC3 (Fig 3B). This suggests that the missense mutation affects the thermal stability of CCDC39, which in turn leads to reduction of N-DRC and axonemal proteins as observed in the null *CCDC39* mutant (Fig 3A).

At 21°C, the *fla12* mutant assembles slightly shorter flagella (~6.4 μm) when compared to wild-type (~8.5 μm). Eight hours after the temperature shift from 21°C to 32°C, the average flagellar length of *fla12* is ~1.6 μm. The flagellar length of the *fla12; cnk11-3* double mutant (~6.5 μm) is similar to the single *fla12* mutant at 21°C. However after temperature shift, the flagellar length is ~3.9 μm, which is significantly longer than *fla12* cells at the same time point (Fig 1B). This indicates that similar to the partial suppression of the short flagella phenotype of *pf7; pf8* double mutant, *cnk11* can partially suppress the temperature-sensitive short flagella phenotype of *fla12*.

**Fig 5:**
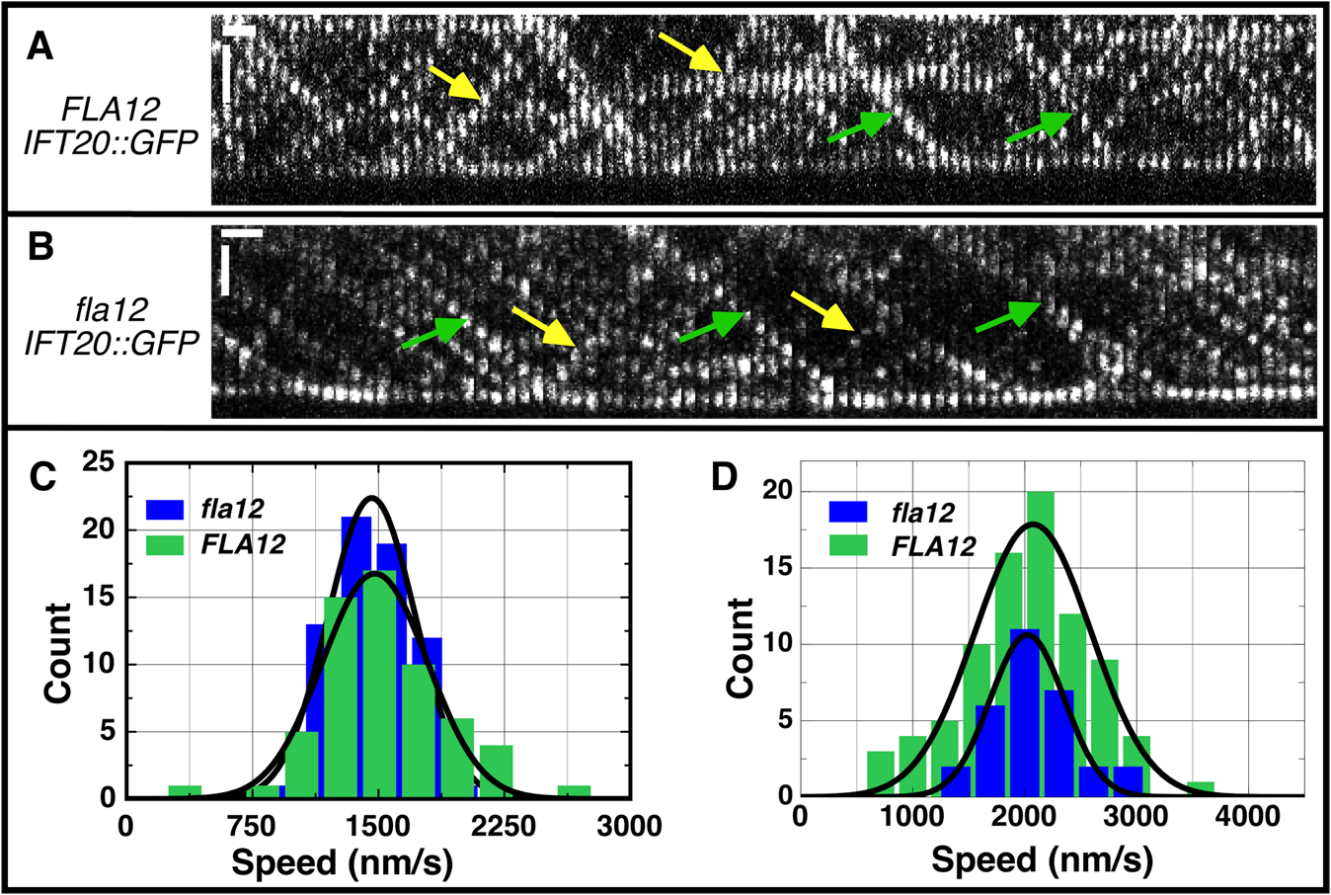
IFT velocities are not changed in the *fla12* mutant. Kymographs of IFT20::GFP in (A) *FLA12* and (B) *fla12* strains. Green and yellow arrows denote representative anterograde and retrograde tracks, respectively. Vertical scale bar, 2 μm; horizontal scale bar, 1 sec. (C) The anterograde IFT velocities were 1478 ± 41 nm/s (mean ± SEM) for *FLA12* cells (green, n = 60) and 1460 ± 32 nm/s for *fla12* cells (blue, n = 67). (D) The retrograde IFT velocities were 2074 ± 56 nm/s for *FLA12* cells (green, n = 84) and 2023 ± 60 nm/s for *fla12* cells (blue, n = 30).

Intraflagellar transport (IFT) was monitored previously in numerous flagellar assembly mutants [58]. Analysis of the *fla12; pf15* double mutant suggested that the velocity of anterograde and retrograde IFT was increased over control velocities, and the frequency of IFT particles was also higher. We reanalyzed IFT by TIRF (Total Internal Reflection Fluorescence) microscopy using GFP-tagged IFT20 [59] in the *fla12* mutant. Data obtained from 4 *FLA12* and 5 *fla12* cells indicate that anterograde (Fig 5C) and retrograde (Fig 5D) IFT velocities in *fla12*; *IFT20-GFP* cells are identical to those in *FLA12*; *IFT20-GFP* cells. Thus, we conclude that there is no IFT velocity defect in the *fla12* mutant. There are at least two possible explanations for the disagreement between these two studies. In the *fla12*; *pf15* study, the *pf15* mutation disrupts the p80 subunit of katanin [60]. It is possible that there is some synthetic interaction between katanin and CCDC39 that affects IFT velocity and number. Alternatively, the multiple backcrosses of *fla12* before the TIRF study could have removed another mutation that affected IFT.

### The *cnk11* mutation suppresses the short flagella phenotype found in N-DRC and dynein arm deficient mutants

**Fig 6:**
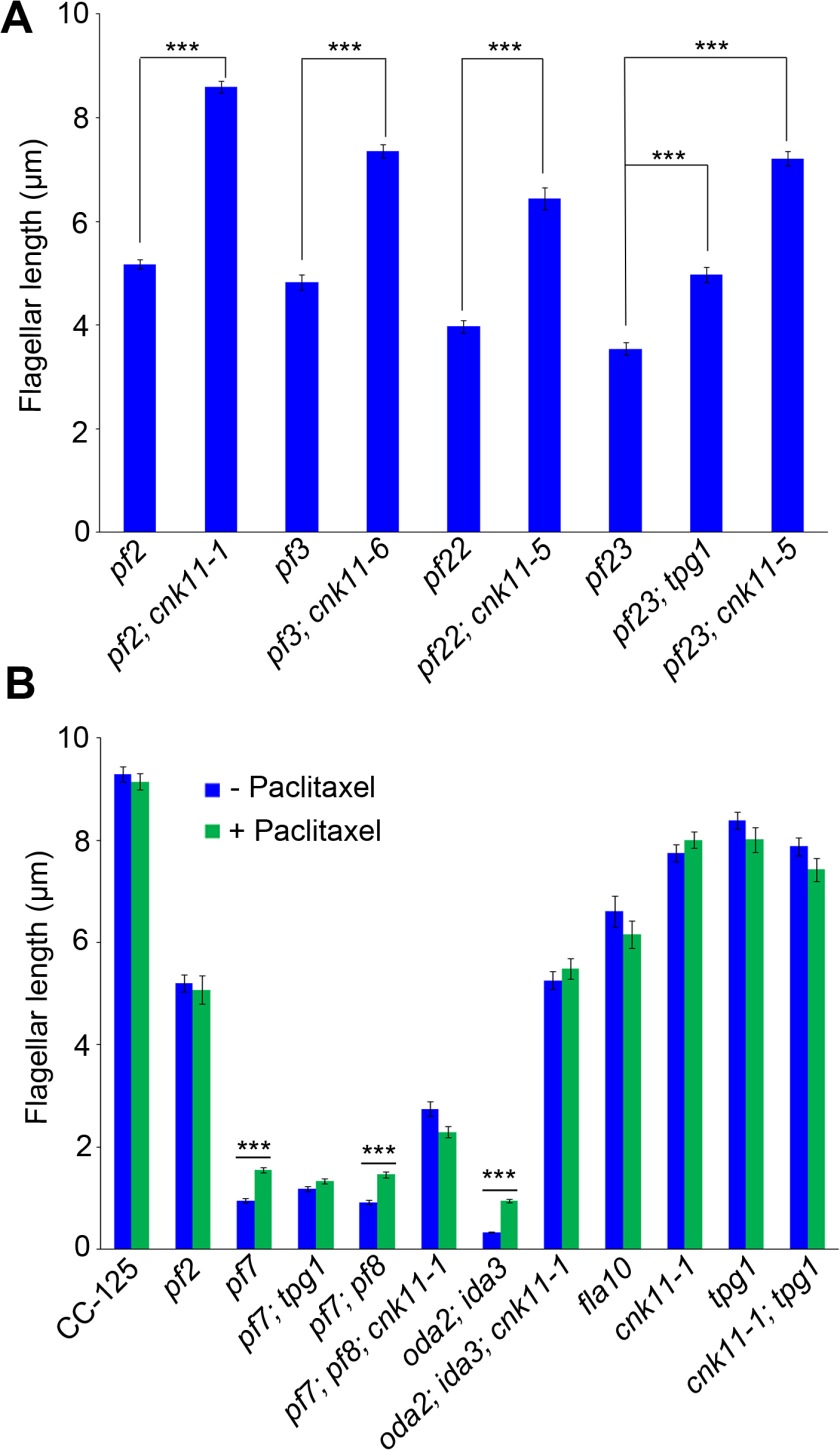
The *cnk11* mutants and paclitaxel can partially rescue flagellar shortness in motility mutants. At least 100 flagella from each strain were measured to determine the average flagellar length. Error bars represent standard deviation of the mean. *** indicates p<0.001 by the t-test. (A) Flagellar length of various strains at 21°C. (B) Cells were either treated with autolysin only (blue) or treated with autolysin and 10 μm paclitaxel (green) for 30 minutes at 21°C before fixation.

To ask about the specificity of the *cnk11* suppressor, we introduced the *cnk11-1* mutation into the *pf2* (DRC4) [10] and *pf3* (DRC1) [61] mutants through meiotic crosses. Mutants in DRC4 are missing N-DRC proteins as well as dyneins *a* and *c* [10]. The *pf2* mutant has an average flagellar length of ~5.2 μm (Fig 6A). In comparison, the *pf2*; *cnk11-1* double mutant has an average flagellar length of ~8.6 μm (Fig 6A), which is comparable to the average flagellar length found in wild-type CC-125 cells (~8.9 μm; Fig 1A) and significantly longer than *pf2* flagella. The *pf3* mutant obtained from the *Chlamydomonas* Resource Center (CC-1026) has an average flagellar length of 7.4 μm (Fig 6A), slightly shorter than in wild-type cells. Mutants in DRC1 are missing N-DRC proteins, tektin, and RSP13 [10]. PCR on progeny from a meiotic cross between CC-1026 and *cnk11-1* revealed that over 8 kb of genomic DNA on chromosome 7 is deleted. The deleted region includes most of the *CNK11* gene and at least half of the adjacent gene Cre07.g339104 (Fig 2). Therefore, the strain CC-1026 should be annotated as *pf3*; *cnk11-6*. A meiotic cross between *pf3*; *cnk11-6* to wild-type CC-124 cells allowed the isolation of a *pf3; CNK11* strain. The average flagellar length of *pf3*; *CNK11* cells is ~4.8 μm (Fig 6A), which is comparable to the length observed in *pf2* cells. In addition, the *pf3; CNK11* cells have flagellar lengths that are more variable (ranging from <1 μm to >8 μm) than the *pf2* (mostly 3~7 μm), *pf7*, and *pf8* (both mostly 1~3 μm) cells (S2 Fig). In conclusion, the *cnk11* mutant rescues the short flagella phenotype of *CCDC39* and *CCDC40* mutants as well as two N-DRC mutants.

The *pf22* and *pf23* mutants were first isolated as paralyzed flagella mutants and both have short flagella [62]. The *PF22* gene encodes a conserved cytoplasmic protein (DNAAF3) that is essential for the assembly of both outer and several inner dynein arms [4]. The *pf23* mutant lacks inner dyneins *a*, *c*, *d*, and *f* [25, 63]. The outer dynein arm mutant, *oda2*, and inner dynein arm mutant, *ida3*, both display slow motility with normal flagellar lengths. The *oda2*; *ida3* double mutant is paralyzed with very short flagella (Fig 6B) as has been observed for many *oda*; *ida* double mutants [26]. The N-DRC is not affected in any of these mutants. To ask whether the *cnk11* suppressor can restore normal flagellar length in these mutants, we introduced *cnk11* mutations into *pf22*, *pf23*, and *oda2*; *ida3* mutants. The average flagellar length of *pf22* is ~4.0 μm and ~6.4 μm for *pf22*; *cnk11-5* (Fig 6A). The average flagellar length of *pf23* is ~3.5 μm and ~7.2 μm for *pf23*; *cnk11-5* (Fig 6A). The *oda2*; *ida3* cells have an average flagellar length of ~0.5 μm; and, the *oda2*; *ida3*; *cnk11-1* triple mutant has an average flagellar length of ~5.9 μm (Fig 6B). Thus, *cnk11* mutations rescue the short flagella mutant phenotype of dynein arm deficient mutants, which lack multiple axonemal dynein species and presumably have unstable axonemal microtubules. Similar to the effect of *cnk11* on *pf7* and *pf8*, the *cnk11* mutations do not rescue the motility defects found in the dynein arm deficient mutants. In addition, the *cnk11* mutations do not rescue the temperature-sensitivity flagellar assembly of the kinesin-2 motor mutant, *fla10*, or the IFT mutants, *fla15* and *fla17*, after 8 hrs at 32°C.

### Chemical suppression of the short flagellar length phenotype

In human cell lines, knockdown of NEK4, a NIMA-like kinase, confers paclitaxel resistance and show defects in repolymerizing microtubules after nocadozole treatment [37]. In *Arabidopsis thaliana*, *nek4*, *nek5*, and *nek6* all show hypersensitivity to paclitaxel [38]. Various NEK proteins play a role in microtubule stability. We tested the *cnk11-1* allele on paclitaxel media with concentrations from 5 to 60 μM. The mutant strain behaved identically to the wild-type controls and we did not observe resistance or hypersensitivity as judged by cell division and cell size [64].

We then asked whether the addition of 10 μM paclitaxel for 30 minutes, a dosage that does not causes arrest of cell division in wild-type cells [64], would have any effect on flagellar length. In wild-type and *cnk11-1* cells, which have normal flagellar lengths, there is no change (Fig 6B). We examined *pf2*, and the temperature-sensitive kinesin mutant *fla10-1* which has about half-length flagella when grown at 28°C [19]. The addition of paclitaxel has no effect on either mutant (Fig 6B). In the short flagella mutants *pf7*, *pf7*; *pf8*, and *oda2*; *ida3*, paclitaxel conferred increased flagellar length. In contrast, paclitaxel does not lead to further elongation of flagella of these mutants when the *cnk11* mutation is present (Fig 6B). This suggests that CNK11 and paclitaxel could act via the same mechanism to stabilize axonemal microtubules in these short flagella mutants.

### The *tpg1* mutant fails to suppress the short flagella phenotype in the *CCDC40* mutant

Kubo *et al*. showed that both *tpg1* and *tpg2* can rescue the short flagella phenotype found in *pf23* and *pf28; pf30* [27]. *PF28* is an allele of *ODA2* (the gamma dynein heavy chain) and *PF30* is an allele of *IDA1 (*1-alpha dynein heavy chain, I1/f complex). This result is similar to the effect of *cnk11* on *pf22*, *pf23*, and *oda2*; *ida3* (Fig 6). Therefore, we asked whether the *tpg1* mutation can rescue the short flagella phenotype found in the *pf7*; *pf8* double mutant. The *TPG1* gene maps to chromosome 17 at 0.51 Mb, between the *PF7* (chromosome 17 at 0.33 Mb) and *PF8* (chromosome 17 at 0.74 Mb) genes. The short distance among these three genes makes it extremely hard to generate the *pf7*; *pf8*; *tpg1* triple mutant by meiotic recombination since it would require two crossovers in an interval of only 4 map units. Given the *pf7* mutant behaves similarly to the *pf7*; *pf8* double mutant, we analyzed *pf7* and *pf7*; *tpg1* instead of *pf7*; *pf8* and *pf7*; *pf8*; *tpg1*.

To our surprise, the *tpg1* mutation does not rescue the short flagella phenotype found in *pf7*. Instead, the *pf7*; *tpg1* mutant has a more severe flagella phenotype than the *pf7* mutant. In nitrogen-free medium, ~85% of *pf7* cells have flagella. In contrast, only ~48% of *pf7*; *tpg1* cells have flagella. Measurement of flagellated cells in both strains showed no significant difference in the flagellar lengths between *pf7* and *pf7*; *tpg1* (Fig 6B).

**Fig 7:**
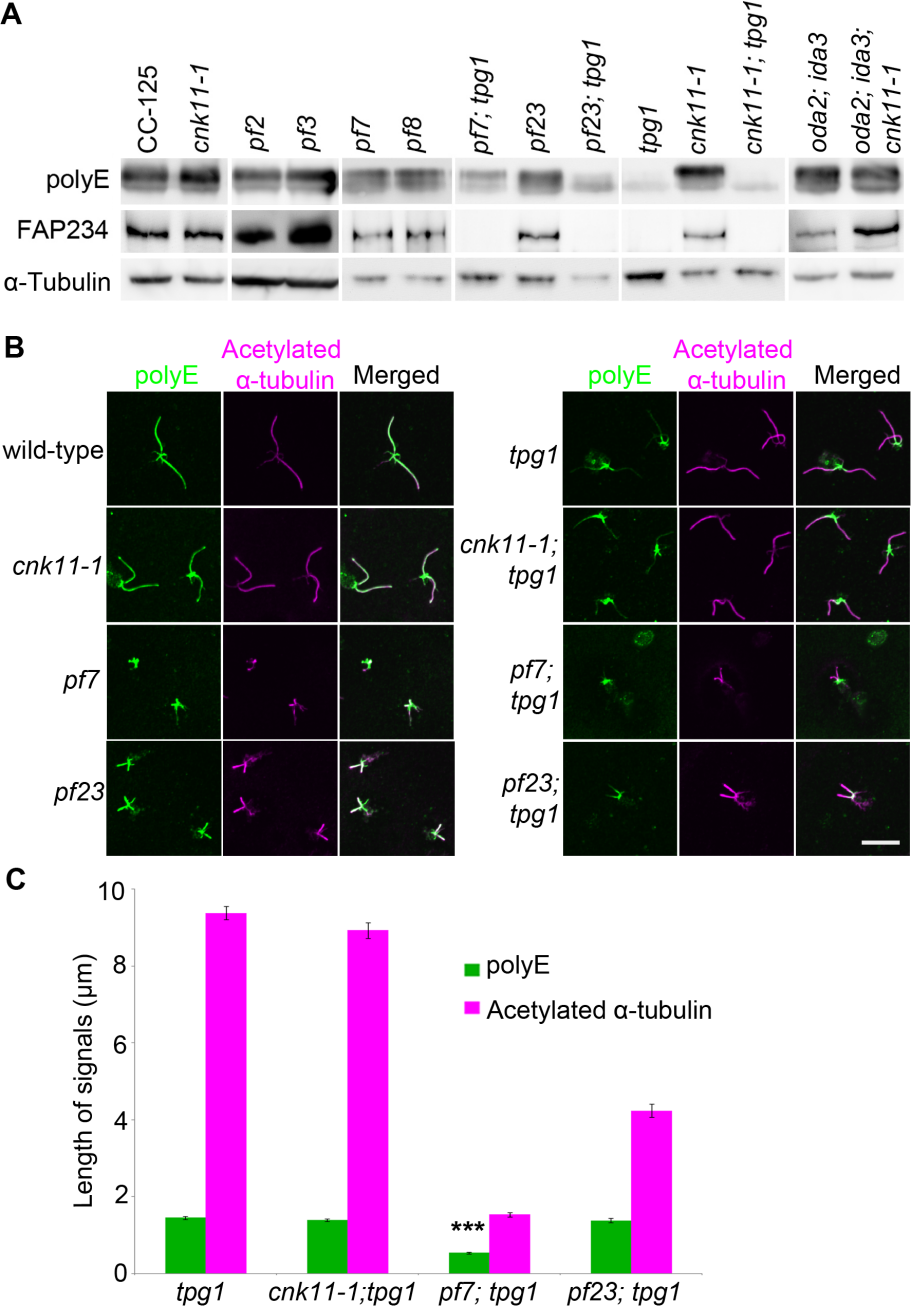
The localization of polyglutamylated tubulin is affected by both *tpg1* and *pf7*. (A) Two micrograms of flagellar protein were used in immunoblots. α-tubulin is included as a loading control. (B) The nucleoflagellar apparatus (NFAP) from various strains was stained with polyglutamylated tubulin antibody (green) and acetylated α-tubulin (magenta). (C) The length of polyglutamylated tubulin (green) and flagella (indicated by acetylated α-tubulin, magenta). *** indicates p<0.001 by the t-test between the lengths of polyglutamylated tubulin in *tpg1* and in *pf7*; *tpg1*.

We asked whether polyglutamylation of tubulin is altered in the *pf7*; *tpg1* mutant by both immunoblots and immunofluorescence. In wild-type cells, tubulin in axonemal microtubules is polyglutamylated. A polyclonal antibody (Poly E) that specifically recognizes tubulin with three or more glutamates reveals much stronger signal intensity in α-tubulin than in β-tubulin in wild-type axonemes [29]. The signal intensity of polyglutamylated α-tubulin relative to polyglutamylated β-tubulin is significantly reduced in *pf7* and *pf8* (Fig 7A). Similar to the findings by Kubo *et al*. [29], we noticed significant reduction of α-tubulin polyglutamylation but not β-tubulin polyglutamylation in *tpg1*. A significant reduction of α-tubulin polyglutamylation was observed in both *pf23*; *tpg1* and *cnk11-1*; *tpg1* mutants, but not in the *pf23* or *cnk11-1* mutants. However in the *pf7*; *tpg1* mutant, polyglutamylated α-tubulin remains (Fig 7A). The change of relative signal intensities between polyglutamylated α- and β-tubulins found in *pf7*, *pf8*, and *pf7*; *tpg1* is not due to their short flagellar lengths, since the short flagellar length mutant *oda2*; *ida3* has stronger signal intensity in α-tubulin than in β-tubulin, as found in wild-type axonemes (Fig 7A). Immunoblots with an anti-TPG2/FAP234 antibody [28] show that no TPG2 protein is detected in the axonemes of any strain carrying the *tpg1* mutant (Fig 7A). It suggests that the presence of small amount of polyglutamylated α-tubulin in *pf7*; *tpg1* is not due to the recruitment or recovery of the TPG1-TPG2 complex in the axoneme. The abundance of TPG2/FAP234 is not significantly affected by flagellar length or the *pf7* and *pf8* mutations.

By immunofluorescence, the polyglutamylated tubulin detected by the polyE antibody shows signal along the entire length of the axoneme in wild-type and *cnk11-1* cells (Fig 7B). As observed previously, the signal in *tpg1* is concentrated at the proximal end [29], and we observe that the polyglutamylated tubulin signal is only ~1.5 μm in length (Fig 7C). The *cnk11-1*; *tpg1* and *pf23*; *tpg1* double mutants have a similar stretch of polyglutamylated tubulin signal regardless of their flagellar lengths (Fig 7B). The *pf7*; *tpg1* cells are strikingly different, the polyglutamylated tubulin signal is reduced to ~0.5 μm, which is significantly shorter than in the single or other double mutants (Fig 7B & 7C). This result is different from what we observed in the immunoblots, in which the polyE signal of α-tubulin is more abundant in *pf7*; *tpg1* than in *tpg1*. It is likely that the difference is due to using isolated flagella that include both the microtubule axoneme and the flagellar membrane/matrix for the immunoblot and using axonemes that have the membrane/matrix fraction removed by detergent for immunofluorescence. Polyglutamylation of α-tubulin but not β-tubulin is associated with soluble tubulin heterodimers [65]. Thus the difference in polyE abundance between the two techniques is likely due to the removal of the soluble polyglutamylated α-tubulin in the immunofluorescence experiments. Combining the immunoblot and immunofluorescence results suggests that PF7/CCDC40 is needed for polyglutamylation at the proximal end of the microtubule axonemes.

Next we asked whether paclitaxel has any effect on *tpg1*. As might be expected for flagella with normal length, neither *tpg1* nor *cnk11-1*; *tpg1* is affected by treatment with paclitaxel for 30 minutes (Fig 6B). However, no increase in flagellar length is observed after paclitaxel treatment of the *pf7*; *tpg1* mutant. We suggest that paclitaxel does not increase flagellar length in strains with the *cnk11* or *tpg1* mutations.

### The *cnk11-1* mutation increases tubulin turnover at the flagellar tip

**Fig 8:**
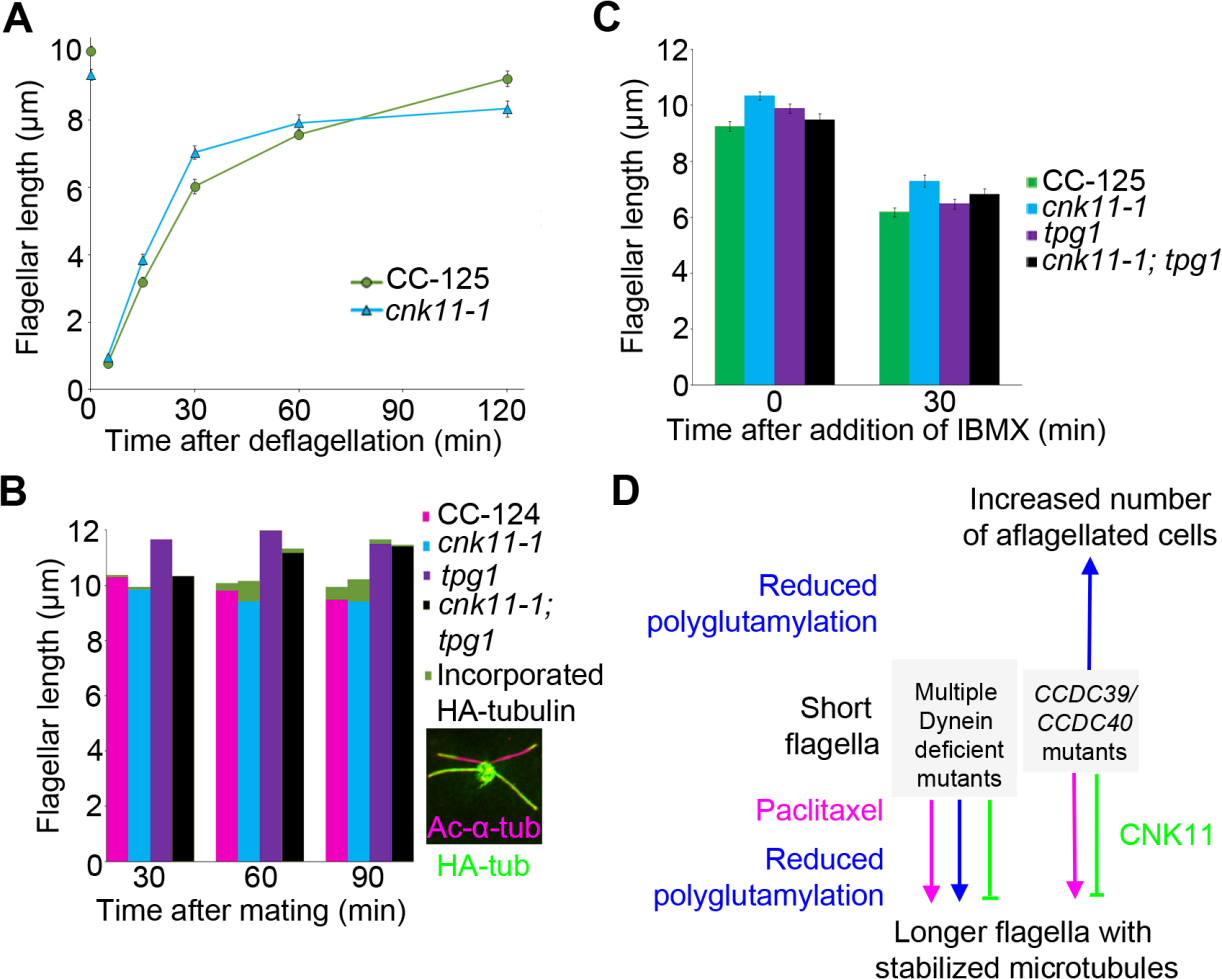
The *cnk11-1* mutant has increased tubulin turnover at the flagellar tip. At least 100 flagella from each strain were measured to determine the average flagellar length. Error bars represent standard deviation of the mean. (A) Flagellar lengths of wild-type (CC-125, green circles) and *cnk11-1* (blue triangles) cells before and after deflagellation by pH shock. (B) Cells that carry HA-α-tubulin were mated to cells of opposite mating type that have the corresponding genotype for 30, 60, and 90 minutes before fixation. Flagellar lengths were measured from the non HA-tagged parental strain in mating of wild-type x wild-type (CC-124, magenta), *cnk11-1* x *cnk11-1* (blue), *tpg1* x *tpg1* (purple), and *cnk11-1*; *tpg1* x *cnk11-1*; *tpg1* (black). The length of new HA-α-tubulin incorporation (green) was measured at the distal end of the flagella. Insert, a representative image of a *cnk11-1* quadriflagellate cells (QFCs) 60 minutes after mating. (C) Flagellar lengths of wild-type (CC-125, green), *cnk11-1* (blue), *tpg1* (purple), and *cnk11-1*; *tpg1* (black) before and 30 minutes after IBMX treatment. (D) A model of flagellar length regulation by paclitaxel (magenta), polyglutamylation (blue), and CNK11 (green). In short flagella mutants caused by multiple dynein deficiency, addition of paclitaxel and reduction of polyglutamylation, as well as blockage of CNK11, leads to longer flagella, presumably due to stabilized axonemal microtubules. In the *CCDC39* and *CCDC40* short flagella mutants, addition of paclitaxel and blockage of CNK11 lead to longer flagella. However, reduced polyglutamylation enhances the mutant phenotype and cause increased number of aflagellated cells.

Given that the NIMA-related kinase *cnk2-1* mutant affects the disassembly rate of flagella, we asked whether *cnk11-1* affects the rates of assembly and/or disassembly. We first compared the rates of flagellar assembly after flagella amputation by pH shock in wild-type (CC-125) and *cnk11-1* cells (Fig 8A). Within 30 minutes following flagellar amputation, the assembly rate of *cnk11-1* cells was ~0.23 μm/min, which is not significantly faster than the rate of wild-type cells (~0.20 μm/min). This is very similar to rates observed in the *cnk2-1* cells by Hilton *et al*. [36]. However, the assembly rate in *cnk11-1* cells reduced significantly within the next 90 minutes, and resulted in slightly shorter flagella than in wild-type cells (Fig 8A). We conclude that the overall assembly rate during flagellar regeneration is not affected in *cnk11-1* cells.

Another way to measure the dynamic of flagellar assembly is to test the incorporation rate of new tubulin subunits at the flagellar tip. When a pair of *Chlamydomonas* cells mate, they form a quadriflagellate cell (QFC), which has two pairs of flagella. Tubulin subunits are added at the tip of the flagella, using subunits from the cytoplasm [19]. The two pairs of flagella can be distinguished by using one parent that carries an epitope-tagged HA-tubulin gene (Fig 8B insert, green), while the other parent lacks this gene. Both pairs of flagella are visualized with an antibody to acetylated α-tubulin (Fig 8B insert, magenta). The flagella from the parent with the tagged α-tubulin are stained with an antibody to the HA tag. Newly incorporated tubulin on the unlabeled flagella is visualized with the antibody to the HA tag. We mated two wild-type strains and tracked the incorporation of HA-tubulin subunits at 30, 60, 90 minutes after mating (Fig 8B, magenta). The length of incorporated HA-tubulin at the tip of flagella gradually increased along time and reached ~0.48 μm at 90 minutes. We mated two parents with the *cnk11-1* mutation and observed more incorporation of HA-tubulin subunits at 60 and 90 minutes (Fig 8B, blue). The length of incorporated HA-tubulin at the tip was ~0.80 μm at 90 minutes, which suggests a rate that is nearly twice as fast as in wild-type QFCs. Since the length of the flagella did not increase, we suggest that the *cnk11-1* mutation increases tubulin turnover at the flagellar tip.

Kubo *et al*. showed that the *tpg2* mutant has slow tubulin turnover at the flagellar tip [27]. We performed the same assay on the *tpg1* mutant. The incorporation length of HA-tubulin in *tpg1* cells was ~0.16 μm at 90 minutes, significantly lower than that in wild-type or *cnk11-1* cells (Fig 8B, purple). The incorporation length of HA-tubulin in *cnk11-1*; *tpg1* cells was ~0.17 μm at 60 minutes but dropped to ~0.05 μm at 90 minutes (Fig 8B, black). Therefore, the faster turnover rate of HA-tubulin observed in *cnk11-1* flagella is suppressed by the *tpg1* mutation.

The addition of 1-isobutyl-3-methylxanthine (IBMX) causes gradual disassembly of flagella in wild-type cells. To ask whether flagellar disassembly is affected by *cnk11*, we compared the flagellar shortening rates in wild-type (CC-125) and *cnk11-1* cells (Fig 8C). The disassembly rates of CC-125 and *cnk11-1* cells within the first 30 minutes were both ~0.10 μm/min, similar to the rate Hilton *et al*. reported [36]. The disassembly rate of *tpg1* (~0.11 μm/min) was similar to wild-type and *cnk11-1*. It was slightly reduced in *cnk11-1*; *tpg1* (~0.09 μm/min, Fig 8C). Thus, neither *cnk11-1* nor *tpg1* mutation affects the flagellar disassembly rate.

## Discussion

Regulation of ciliary and flagellar length is extremely important to the proper function of these organelles in different organisms. In humans, ciliary length defects are observed in multiple ciliopathies that include Bardet-Biedl syndrome, nephronophthisis, Joubert syndrome, polycystic kidney disease, and Meckel-Gruber syndrome [66]. In *Chlamydomonas*, flagellar length defects affect both motility and mating [67]. Flagellar length can be regulated by multiple factors, including the rates of flagellar assembly and disassembly [66, 68], availability of IFT proteins, motors, and structure proteins [20], as well as factors that affect the stability of axonemal microtubules [69].

### The structure defects in *CCDC39* and *CCDC40* mutants

In mutant screens performed by McVittie and others, three *pf7* and five *pf8* alleles were identified [17, 70]. The mutants show abnormalities in the organization of the axoneme and radial spokes [17]. Oda and colleagues localized CCDC39 and CCDC40 using tagged genes together with cryo-EM tomography to show that these proteins serve as docking sites along the doublet microtubules for axonemal structures, which include the radial spokes, the N-DRC and all of the inner dynein arms [16]. Our immunoblots with DLE2/centrin suggest that not all of the inner arms are missing in *pf7* and *pf8* since centrin associates with three inner dynein heavy chains (*b*, *e*, and *g*) and is only slightly reduced. Although the *pf7* and *pf8* mutants have paralyzed flagella, their dyneins are functional based on our sliding/splaying assays. Both single mutants and the double mutant show splaying of the microtubules (Fig 4) that is similar to the splaying observed in the N-DRC mutants [10]. Thus, the paralysis is likely to be due to the microtubule and radial spoke disorganization that regulate the coordinated behavior of the dynein arms, as hypothesized by both McVittie and Oda *et al*. [16, 17]. The splaying experiments also suggest that the link in the proximal 2 μm does not rely on CCDC39/40, DRC1, DRC4, or the inner dynein arm I1/f (Fig 4 and [10]). Bui and colleagues [71] identified rod-like circumferential interdoublet linkers in the proximal axoneme that are clearly structural different from the N-DRC structure. We assume that these structures are retained in the *pf7* and *pf8* mutants, but they have not been examined.

In all patients diagnosed with PCD with *CCDC39* or *CCDC40* mutations, the changes result in premature truncation of the protein, which suggests that null alleles are associated with the phenotype [11]. Unexpectedly, the long-term prognosis of children with *CCDC39* or *CCDC40* mutations is worse than for other PCD patients, and similar to patients with cystic fibrosis [18]. These alleles would be similar to the mutations in *pf7* and *pf8* that have premature termination alleles. We also identified a conditional allele, *fla12*, in the *PF8/CCDC39* locus (Table 1). The leucine to proline change occurs in an unstructured region of the C-terminus of the protein and the leucine is not conserved in other organisms. At the permissive temperature (21°C) the flagella are slightly shorter. This missense mutation leads to reduced CCDC39 and other DRC proteins at the restrictive temperature (Fig 3B). After 8 hours at the restrictive temperature, the phenotype of *fla12* cells resembles the phenotype of *pf8* cells. The flagella are immotile and short. It is possible that missense alleles in *CCDC39* in humans may have a less severe phenotype that only slightly alters the motility and would not have been grouped together with the more severe null alleles associated with PCD [11, 12, 14]. Our *fla12* revertants (Table 3) indicate that the leucine can be replaced by a variety of amino acids. This suggests that the change to a proline undermines the protein function and leads to the short and paralyzed flagella at 32°C. Given that the speeds of anterograde and retrograde IFT in *fla12* shows no difference compared to those found in wild-type cells, it suggests that IFT is unlikely to play a role in flagellar length control in this *CCDC39* mutant.

### The polyglutamylation defect in *CCDC39* and *CCDC40* mutants

Post-translational modification of tubulin, which includes polyglutamylation and polyglycination, affects axonemal microtubule stability. Suryavanshi *et al*. and Kubo *et al*. showed in *Tetrahymena* and *Chlamydomonas* that loss of polyglutamylation on the B-tubule is likely to affect the activity of inner arm dyneins [72, 73]. A decrease in tubulin polyglutamylation in mouse airway cilia changes the curvature of the cilia as well as the asymmetry of the beating [74]. Overexpression of the polyglutamylation enzyme (TTLL6) in *Tetrahymena* destabilizes axonemal microtubules [75]. Knockdown of the glycination enzymes (TTLL3 and TTLL8) causes instability and results in short or absent mouse ependymal cilia. Polyglutamylation changes the binding affinities of a number of microtubule associated proteins and motors [76], and promotes microtubule severing [77]. Thus, the presence of polyglutamylation may affect microtubule stability in a variety of ways. Polyglutamylation like acetylation of tubulin is associated with long-lived microtubules [76, 78].

Because the loss of CCDC39 and CCDC40 affects the level of polyglutamylation, we examined the *tpg1* mutation in *TTLL9*. Loss of α-tubulin polyglutamylation in *tpg1* causes a motility defect due to the loss of tektin but no change in flagellar length ([29] and Fig 6B). Thus, reduction in tubulin polyglutamylation in the *pf7* and *pf8* mutants cannot be solely responsible for the short flagella in the CCDC39/40 mutants. The *tpg1* mutation in combination with either *pf7* or inner dynein arm deficient mutant *pf23* has very different consequences. The *tpg1* mutant partially rescues the flagellar length defect in *pf23* (Fig 6A) but leads to more aflagellate cells with *pf7* and no change in the length of the remaining flagella. By immunoblots, the level of polyglutamylated α-tubulin in the flagella of *pf23*; *tpg1* is significantly less than in the flagella of *pf7*; *tpg1* (Fig 7A). By immunofluorescence, we show that localization of polyglutamylated tubulin at the proximal end of axoneme is reduced in *pf7*; *tpg1*, but not in *pf23*; *tpg1*, when compared to *tpg1* (Fig 7B & 7C). One possibility is that CCDC39 and CCDC40 are required for the activity of one or more tubulin glutamylases at the proximal end of the flagella while the TPG1 is responsible to polyglutamylation of tubulin along the rest of the flagella. There are 10 TTLL proteins found in *Chlamydomonas* [29] and the flagellar proteome includes only the TTLL9/TPG1 protein [79]. However, a proteomic analysis of flagellar phosphoproteins indicates that at least 3 additional TTLL proteins are found in the flagella [80]. They include TTLL13, a homolog of human tubulin polyglutamylase TTLL6; Cre09.g403108, an ortholog of human tubulin polyglutamylases TTLL4 and TTLL5; and Cre03.g145447, a homolog of human monoglycylase TTLL3. The former two are good candidates to be involved in tubulin glutamylation in the flagella.

### The involvement of protein kinases in flagellar length control

Multiple protein kinases affect flagellar length. In *Chlamydomonas*, three CDK-like kinases (LF2, LF5, and FLS1), one MAP kinase (LF4), and one NIMA-related kinase (CNK2), have been characterized [36, 57, 81–83]. Loss of LF2, LF4, LF5, and CNK2 increase flagellar length. Loss of FLS1, CNK2, and LF4 block flagellar disassembly and loss of CNK2 decreases incorporation of new tubulin at the flagellar tip. The direct targets of these kinases remain to be identified. A recent global phosphoproteomic study revealed that over 180 *Chlamydomonas* flagellar proteins are phosphorylated [80]. This set includes N-DRC proteins, IFT proteins, outer and inner dynein arm proteins, central pair proteins, radial spoke proteins, and CCDC39.

Our screen for suppressors of the *pf7*; *pf8* double mutant was designed to find swimming cells. However, no restoration of motility was found. The *cnk11* mutant alleles led to short stumpy flagella, which still have a motility defect. In addition, we found that the *cnk11* mutant alleles rescue the flagellar length defect but not the motility defect of N-DRC mutants as well as the dynein arm deficient mutants (Fig 6A). These results, along with the fact that multiple DRC proteins and axonemal proteins are not restored in *pf7*; *pf8*; *cnk11-1* and *pf3*; *cnk11-6* mutants (Fig 3A), suggest that the *cnk11* mutations partially increase flagellar length via a N-DRC- and dynein protein-independent mechanism. Thus, even though CCDC39 is found to be phosphorylated in the flagella [80], it is unlikely that it is the direct target of CNK11.

During flagellar assembly, a cytoplasmic pool of tubulin subunits are constantly transported to the tip of flagella via IFT [59]. Different from flagellar assembly, flagellar disassembly is not dependent on flagellar length [19]. It is affected by the rates of IFT, disassembly of axonemal microtubules, and disassembly of axoneme-associated protein [81]. Unlike the *lf2*, *lf4*, *lf5*, and *cnk2* mutants, both *cnk11-1* and *tpg1* mutants have normal flagellar length (Figs 1A and 6B and [29]), flagellar assembly (Fig 8A and [29]), and flagellar disassembly (Fig 8C). One difference between *cnk11-1* and *tpg1* mutants is that *cnk11-1* shows a faster than normal tubulin turnover rate at the flagellar tip and *tpg1* has a slower than normal rate (Fig 8B). The double mutant shows a turnover rate similar to *tpg1* (Fig 8B) and proximal end localized polyglutamylated tubulin similar to *tpg1* (Fig 7B & 7C). It is unlikely that TPG1 and TPG2 are the direct targets of CNK11, given that they are not found in the flagellar phosphoproteome [80].

In conclusion, we found that *CCDC39* and *CCDC40* mutants that have short flagella and fail to assemble the N-DRC and several inner dynein arms. Post-translational modification such as polyglutamylation and phosphorylation can affect flagellar length via IFT-independent and structural protein-independent pathways. These modification, may function similarly to the microtubule stabilizing drug paclitaxel and stabilize the unstable axonemal microtubules found in short flagella mutants. Further analysis of other short flagella mutants, such as *shf1*, *shf2*, and *shf3* [84], or *pf21* [85], are likely to identify more genes involved in flagellar assembly and length control.

## Materials and Methods

### Strains and culture conditions

The *pf7* and *pf8* mutant strains were obtained from the Chlamydomonas Resource Center as CC- 568 and CC-560. The strains from the stock center were aflagellate. Different media conditions were tried, but less than 20% of the cells assembled flagella. Both mutants were backcrossed to CC-124 three successive times to determine if reassorting the genome would increase the fraction of flagellated cells. After three backcrosses, strain *pf8 2–4* was chosen. After 4 hours in nitrogen-free HSM medium, greater than 10% of cells had ~7 μm flagella. These were used for additional matings and for flagella preparations. After two backcrosses, strain *pf7 2–2* was chosen. After 4 hours nitrogen-free medium, greater than 80% of cells had short (<4μm) flagella. Other strains and culture conditions are as reported previously [86]. Treatment of cells with paclitaxel was performed in yellow Lucite boxes to prevent breakdown of the paclitaxel by white light [64, 87].

### Whole genome sequencing

*Chlamydomonas* genomic DNA preparation for whole genome sequencing was prepared as described previously [86]. Three micrograms of DNA was submitted to Genome Technology Access Core (Washington University School of Medicine) for library construction, Illumina sequencing, and initial data analysis. For multiplex Illumina sequencing, 7-nucleotide indexes were added to individual DNAs during the library construction before the samples were subjected to sequencing.

Illumina whole genome sequencing reads were aligned onto the *Chlamydomonas* version 5.3.1 genome assembly, and then aligned to JGI predicted exomes ([42] for details). SAMTools [88] were used for calling of SNPs/short indels from each strain. The SNPs/short indels from individual strains were compared to a SNP/short indel library (http://stormo.wustl.edu/dgranas/form.php) generated from 16 previously sequenced strains (15 in the cases of *pf7* and *pf8* because they were included as two of 16 original strains used to construct the library) [20, 42]. The unique SNPs/short indels in each strain were analyzed and filtered by SnpEff [89]. Only changes that have Phred quality scores of over 100 and rest within the coding region and splicing sites were retained. Whole genome sequencing reads of *pf7* and *pf8* can be found under NCBI BioProject Accession Number PRJNA245202. Whole genome sequencing reads of *fla12*, *pf7*; *pf8*; *cnk11-1*, *pf7*; *pf8*; *cnk11-2*, *pf7*; *pf8*; *cnk11-3*, *pf7*; *pf8*; *cnk11-4*, and *pf7*; *pf8*; *cnk11-5* can be found under NCBI BioProject Accession Number PRJNA293107.

### Revertant analysis

Revertant analysis was performed as previously reported [86]. Most of the mutant cells fail to oppose gravity and fall to the bottom of the tube. Swimming cells rise to the top of the tube and the upper 10 mL was transferred to fresh medium five times over the course of 13 days. Cultures were plated for individual colonies and one colony with swimming cells was kept from each tube. For the isolation of suppressors of the *pf7*; *pf8* strains, we failed to recover any swimming cells. However, the nature of the pellet changed following the rounds of enrichment. Instead of large clumps of cells, the pellets were smooth and there were single cells. This was used to identify the suppressors.

### Flagellar length measurement

Two day-old cells were resuspended in nitrogen-free medium for 4 hours before treated with freshly made autolysin and fixed in cold methanol. Cells were stained with anti-acetylated α-tubulin antibody followed by Alexa 594-conjugated goat anti-mouse secondary antibody. ImageJ was used to measure the flagellar length.

### Axonemal isolation and immunoblots

Protocols are as described previously [20]. Antibodies used in this study are listed in S4 Table.

### Axoneme reactivation

Cells were deflagellated by pH shock and the isolated flagella were resuspended in demembranating buffer as described [10]. Half of the resultant axonemes were treated with 0.1 mM ATP at room temperature for 4 minutes. Both ATP-treated and non-treated axonemes were fixed with 2% paraformaldehyde at room temperature for 10 minutes on poly-lysine-coated multi-well slides (Thermo Scientific). The slide was then immersed in cold methanol for 10 minutes at -20°C. The samples were allowed to air dry on the slide before the addition of blocking buffer (5% BSA, 1% fish gelatin). The primary antibodies used were LF5 (1:200 dilution) and acetylated α-tubulin (1:250 dilution) diluted in 20% blocking buffer. The secondary antibodies were Alexa 488-conjugated goat-anti-rabbit IgG (1:500 dilution) and Alexa 594-conjugated goat-anti-mouse IgG (1:500 dilution) diluted in 20% blocking buffer.

### TIRF microscopy for IFT velocity measurements

Cells were imaged on manufacturer pre-cleaned fused silica chips (6W675-575 20C, Hoya Corporation USA, San Jose, CA), and sandwiched between the fused silica surface and a coverslip (1.8 x 1.8 cm^2^), resulting in a 25 μm thick water layer that allowed the 10 μm diameter *Chlamydomonas* cell body to move freely in solution. We used total internal reflection fluorescence (TIRF) microscopy to image the cells. The details of the imaging methods were reported previously [90]. Videos of individual surface-attached flagella were processed into kymographs. For visible IFT tracks in a kymograph, a minimum of 3 consecutive and clearly distinguishable IFT20::GFP intensity profiles were required for a track to be used. For each selected IFT track, the slope of the line through the centroid of the first and last IFT20::GFP intensity profiles in the track was used to determine the IFT velocity.

## Supporting Information

S1 Fig Phylogenetic analysis of 77 NIMA-related protein kinases from 12 different organisms. Numbers next to each node were obtained from sampling of 100 bootstrap analyses. *Chlamydomonas* proteins are indicated in green. A magenta arrow in the figure indicates the position of CNK11.
(TIF)

S2 Fig Flagellar length distribution in N-DRC mutants (*pf2* and *pf3*) and *CCDC39* and *CCDC40* mutants. Flagellar length were measured in 100 cells from each mutant and rounded to the nearest integer. Red circles, *pf2*; blue diamonds, *pf3*; yellow squares, *pf7*; green triangles, *pf8*.
(TIF)

S1 Table Linkage of *cnk11* suppressors. (DOCX)

S2 Table dCAPs markers used in this study. (DOCX)

S3 Table Proteins used in the construction of phylogenetic tree shown in S1 Fig. (DOCX)

S4 Table Antibodies used in this study. (DOCX)
