# Supplementary figures and images for: A NIMA-Related Kinase Suppresses the Flagellar Instability Associated with the Loss of Multiple Axonemal Structures
Source: PLoS Genet. 2015 Sep 8;11(9):e1005508. doi: 10.1371/journal.pgen.1005508 (PMC4562644; doi:10.1371/journal.pgen.1005508)

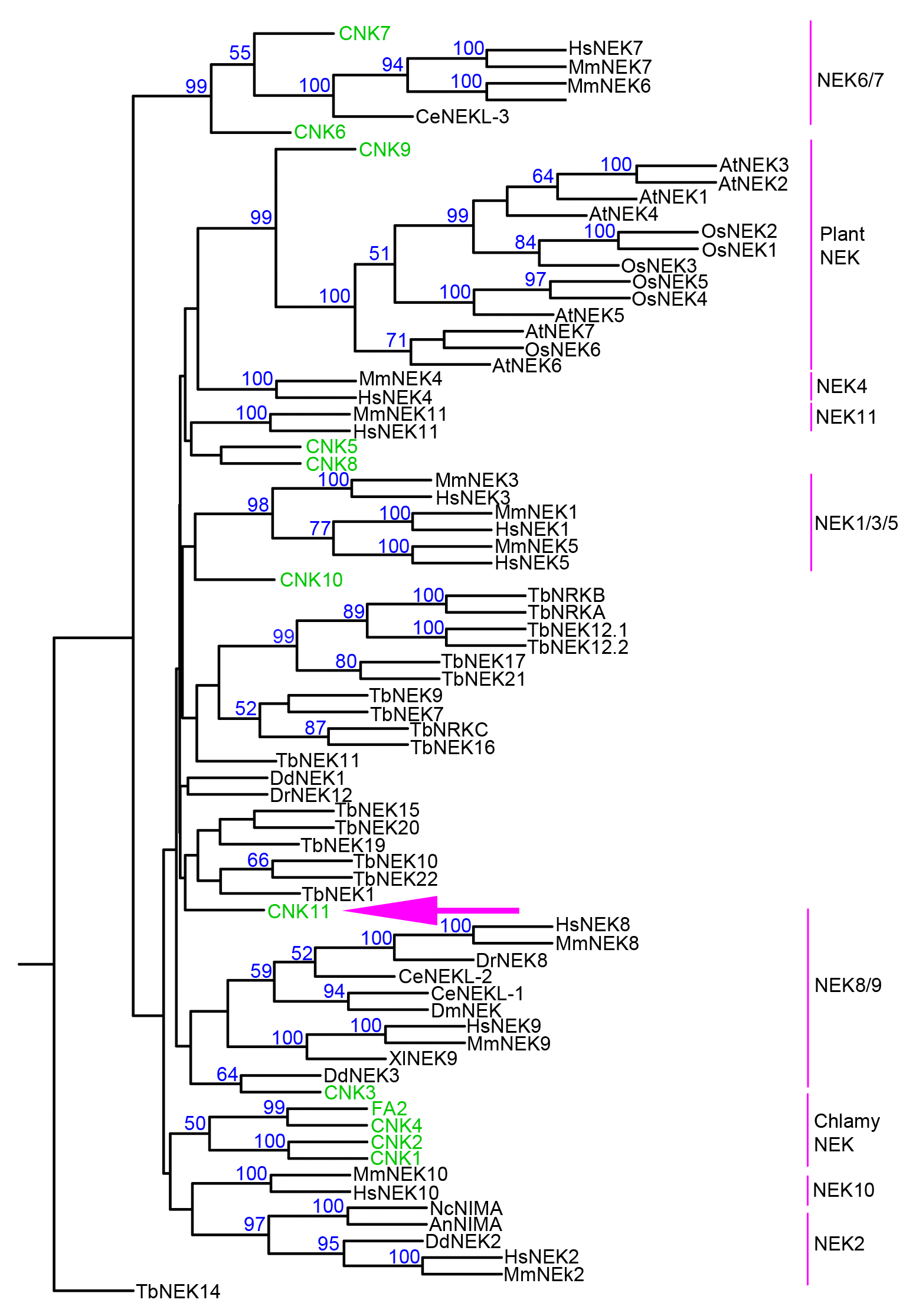

Supplement: S1 Fig — Numbers next to each node were obtained from sampling of 100 bootstrap analyses. Chlamydomonas proteins are indicated in green. A magenta arrow in the figure indicates the position of CNK11. (TIF) [file pgen.1005508.s001.tif]

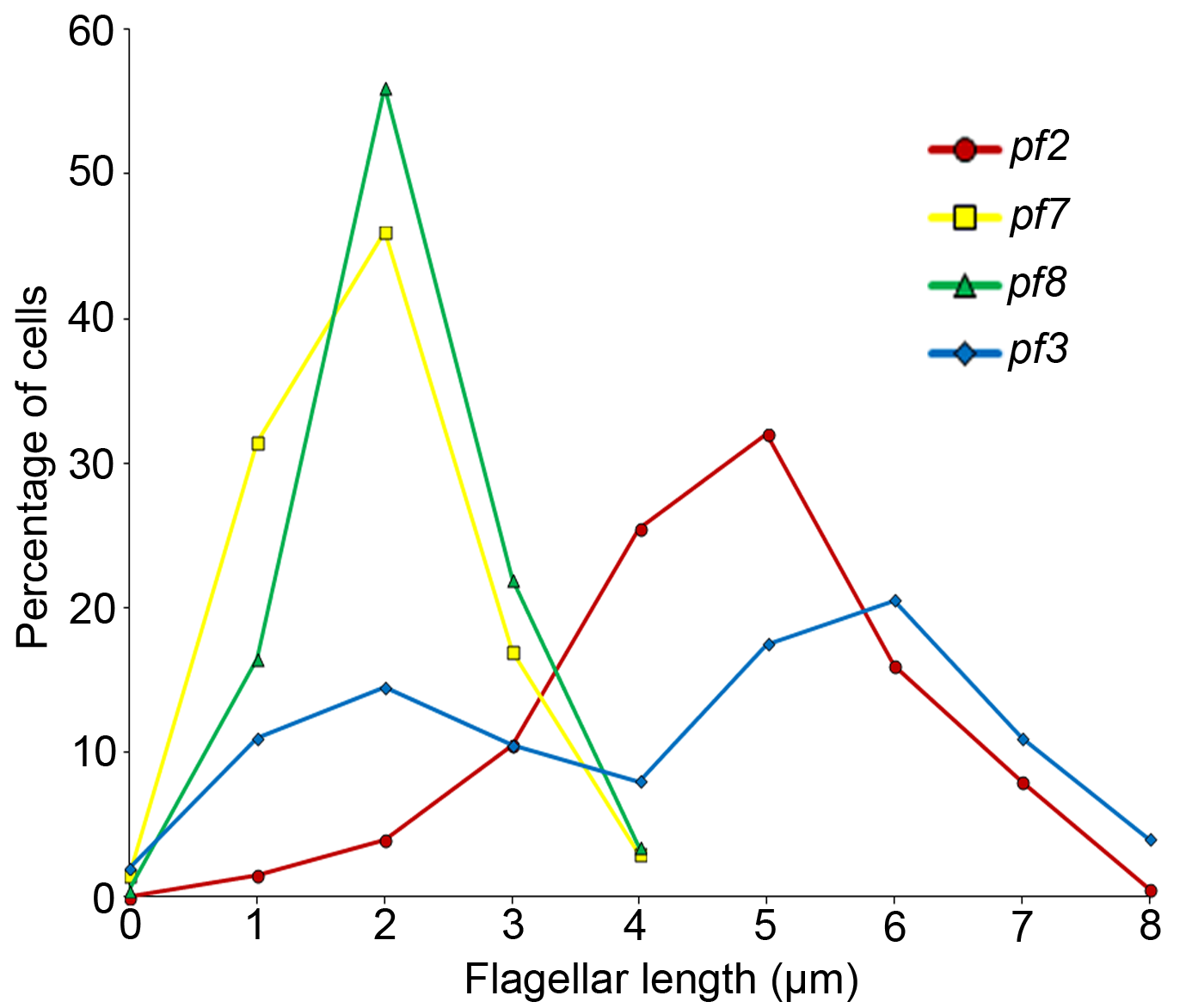

Supplement: S2 Fig — Flagellar length were measured in 100 cells from each mutant and rounded to the nearest integer. Red circles, pf2; blue diamonds, pf3; yellow squares, pf7; green triangles, pf8. (TIF) [file pgen.1005508.s002.tif]
