# Supplementary material for: A NIMA-Related Kinase Suppresses the Flagellar Instability Associated with the Loss of Multiple Axonemal Structures
Source: PLoS Genet. 2015 Sep 8;11(9):e1005508. doi: 10.1371/journal.pgen.1005508 (PMC4562644; doi:10.1371/journal.pgen.1005508)
Supplement: S1 Table — (DOCX) [file pgen.1005508.s003.docx]

**S1 Table: Linkage of *cnk11* suppressors**

| **Mutant** | **wild-type** | ***pf7; pf8*** | ***pf7; pf8; cnk11-2*** | ***pf7; pf8; cnk11-3*** | ***pf7; pf8; cnk11-4*** | ***pf7; pf8; cnk11-5*** |
| --- | --- | --- | --- | --- | --- | --- |
| ***pf7; pf8; cnk11-1*** | 3:3:3 | 12:0:0 | 6:0:0 | 9:0:0 | 12:0:0 | 11:0:0 |
| ***pf7; pf8; cnk11-2*** | 4:3:2 | 13:0:0 | Not tested | 8:0:0 | 6:0:0 | 13:0:0 |
| ***pf7; pf8; cnk11-3*** | 5:3:3 | 11:0:0 | 5:0:0 | Not tested | 8:0:0 | 4:0:0 |
| ***pf7; pf8; cnk11-4*** | 2:4:4 | 12:0:0 | 14:0:0 | 5:0:0 | Not tested | 4:0:0 |
| ***pf7; pf8; cnk11-5*** | 3:4:3 | 15:0:0 | 8:0:0 | 4:0:0 | 7:0:0 | Not tested |
