# Supplementary material for: A NIMA-Related Kinase Suppresses the Flagellar Instability Associated with the Loss of Multiple Axonemal Structures
Source: PLoS Genet. 2015 Sep 8;11(9):e1005508. doi: 10.1371/journal.pgen.1005508 (PMC4562644; doi:10.1371/journal.pgen.1005508)
Supplement: S2 Table — (DOCX) [file pgen.1005508.s004.docx]

**S2 Table: dCAPs markers used in this study**

| **Mutant** | **Forward primer** | **Reversed primer** | **Product sizes**  **(wild-type; mutant)** | **Enzyme** |
| --- | --- | --- | --- | --- |
| *pf7* | CCT ATT CCA GCC CTT CCT TC | GTC TGT GGT GCC AAG GCT TA | 168 + 82 + 51;  250 + 51 | *Pvu*II |
| *pf8* | AGC ATC CCT CGC CTC TTC | TCC AGT TCA TGA GCA TTT TGA | 107 + 63;  170 | *Nla*IV |
| *fla12* | Cgc gcc ttt cca acc tg | Cac gcc tcc gca atc at | 194 + 89; 138 + 89 + 56 | *MspA1*I |
| *cnk11-1* (1A) | GGT GAG ATG AAG GCT CCA AA | CCT CTG GAA TGA CGC TGA G | 150+96+10; 246+10 | *Mnl*I |
| *cnk11-2* (1E) | CAC GCC CTA TTG ACG AAC TAC | CCT AAC CCC TCT TTT GCA TT | 139+47+36; 139+ 83 | *Bsl*I |
| *cnk11-3* (1C)  *cnk11-5* (2B) | GTC GCC AGG CGG AGT GAC | CGT GGT GAT GAG CTG CTG | 209; 117+91 | *Bgl*I |
| *cnk11-4* (2C) | CAC GCC CTA TTG ACG AAC TAC | CCT AAC CCC TCT TTT GCA TT | 107+85+30; 115+107 | *Hae*III |
| *cnk11-6* | ATG CAT ATT TGA CGG GCG TA | CCT TGC GAC TCC ATA GAC TG | 300; 300 | N/A |
| *cnk11-6* | GGT GAG ATG AAG GCT CCA AA | CCT CTG GAA TGA CGC TGA G | 256; 0 | N/A |
| *cnk11-6* | CAC GCC CTA TTG ACG AAC TAC | CCT AAC CCC TCT TTT GCA TT | 222; 0 | N/A |
| *cnk11-6* | GTC GCC AGG CGG AGT GAC | CGT GGT GAT GAG CTG CTG | 259; 0 | N/A |
| *cnk11-6* | TGC GAA ATA GGA TGG CTA CG | TGC TCT TGG GAA AGG CAA AT | 106; 0 | N/A |
| *cnk11-6* | CCG GCG AAA GGT ATT GGT TA | CAT GAA GGT TAG GGT CGT GG | 111; 0 | N/A |
| *cnk11-6* | CAC ACA GGG AGA ACA TCG AG | ACT TGG GTG CAC AAA TCC AT | 277; 0 | N/A |
| *cnk11-6* | CGC ATC TCT GTG CTG TTG TT | CGC TAA TCT CAC TGG TCC CT | 275; 275 | N/A |
