# Supplementary material for: A NIMA-Related Kinase Suppresses the Flagellar Instability Associated with the Loss of Multiple Axonemal Structures
Source: PLoS Genet. 2015 Sep 8;11(9):e1005508. doi: 10.1371/journal.pgen.1005508 (PMC4562644; doi:10.1371/journal.pgen.1005508)
Supplement: S3 Table — (DOCX) [file pgen.1005508.s005.docx]

**S3 Table: Proteins used in the construction of phylogenetic tree shown in S1 Fig**

| **Protein** | **Accession #** | **AA used** | **Organisms** |
| --- | --- | --- | --- |
| AtNEK1 | AEE33112.1 | 4-262 | *Arabidopsis thaliana* |
| AtNEK2 | AEE74138.1 | 4-262 | *Arabidopsis thaliana* |
| AtNEK3 | AED93785.1 | 4-262 | *Arabidopsis thaliana* |
| AtNEK4 | AEE80461.1 | 4-262 | *Arabidopsis thaliana* |
| AtNEK5 | AEE77875.1 | 8-266 | *Arabidopsis thaliana* |
| AtNEK6 | Q9LT35.1 | 4-261 | *Arabidopsis thaliana* |
| AtNEK7 | AEE75165.1 | 17-287 | *Arabidopsis thaliana* |
| AnNIMA | P11837.1 | 9-299 | *Aspergillus nidulans* |
| CeNEKL-1 | NP_490968.2 | 171-430 | *Caenorhabditis elegans* |
| CeNEKL-2 | CCD64899.1 | 4-265 | *Caenorhabditis elegans* |
| CeNEKL-3 | CAA92169.2 | 23-284 | *Caenorhabditis elegans* |
| CrNEK1 | Cre02.g075350 | 16-272 | *Chlamydomonas reinhardtii* |
| CrNEK2 | Cre12.g560350 | 11-266 | *Chlamydomonas reinhardtii* |
| CrNEK3 | Cre06.g268150 | 1-233 | *Chlamydomonas reinhardtii* |
| CrNEK4 | Cre05.g232750 | 9-261 | *Chlamydomonas reinhardtii* |
| CrNEK5 | Cre10.g443300 | 4-260 | *Chlamydomonas reinhardtii* |
| CrNEK6 | Cre12.g514650 | 72-331 | *Chlamydomonas reinhardtii* |
| CrNEK7 | Cre12.g499850 | 57-312 | *Chlamydomonas reinhardtii* |
| CrNEK8 | Cre07.g328850 | 4-260 | *Chlamydomonas reinhardtii* |
| CrNEK9 | Cre02.g107800 | 6-266 | *Chlamydomonas reinhardtii* |
| CrNEK10 | Cre01.g001800 | 4-258 | *Chlamydomonas reinhardtii* |
| CrNEK11 | Cre07.g339100 | 582-921 | *Chlamydomonas reinhardtii* |
| CrFA2 | AAL86904.1 | 17-282 | *Chlamydomonas reinhardtii* |
| DrNEK8 | Q90XC2.1 | 1-192 | *Danio rerio* |
| DrNEK12 | NP_001017548.1 | 4-264 | *Danio rerio* |
| DdNEK1 | Q54QD5.1 | 10-287 | *Dictyostelium discoideum* |
| DdNEK2 | Q55BN8.1 | 4-267 | *Dictyostelium discoideum* |
| DdNEK3 | Q86I06.1 | 4-268 | *Dictyostelium discoideum* |
| DmNEK | AAF56344.1 | 105-363 | *Drosophila melanogaster* |
| HsNEK1 | Q96PY6.2 | 4-258 | *Homo sapiens* |
| HsNEK2 | P51955.1 | 8-268 | *Homo sapiens* |
| HsNEK3 | P51956.2 | 4-257 | *Homo sapiens* |
| HsNEK4 | P51957.2 | 6-261 | *Homo sapiens* |
| HsNEK5 | Q6P3R8.1 | 4-259 | *Homo sapiens* |
| HsNEK6 | Q9HC98.2 | 45-302 | *Homo sapiens* |
| HsNEK7 | Q8TDX7.1 | 34-291 | *Homo sapiens* |
| HsNEK8 | Q86SG6.1 | 7-258 | *Homo sapiens* |
| HsNEK9 | NP_149107.4 | 52-308 | *Homo sapiens* |
| HsNEK10 | Q6ZWH5.3 | 519-785 | *Homo sapiens* |
| HsNEK11 | Q8NG66.2 | 29-287 | *Homo sapiens* |
| MmNEK1 | P51954.2 | 4-258 | *Mus musculus* |
| MmNEK2 | O35942.2 | 9-275 | *Mus musculus* |
| MmNEK3 | Q9R0A5.2 | 4-259 | *Mus musculus* |
| MmNEK4 | Q9Z1J2.1 | 6-265 | *Mus musculus* |
| MmNEK5 | Q7TSC3.1 | 4-259 | *Mus musculus* |
| MmNEK6 | Q9ES70.1 | 45-306 | *Mus musculus* |
| MmNEK7 | Q9ES74.1 | 34-295 | *Mus musculus* |
| MmNEK8 | Q91ZR4.1 | 4-262 | *Mus musculus* |
| MmNEK9 | Q8K1R7.2 | 52-312 | *Mus musculus* |
| MmNEK10 | Q3UGM2.2 | 519-789 | *Mus musculus* |
| MmNEK11 | Q8C0Q4.2 | 30-292 | *Mus musculus* |
| NcNIMA | EAA36051.2 | 7-294 | *Neurospora crassa* |
| OsNEK1 | Q10GB1.1 | 4-262 | *Oryza sativa* |
| OsNEK2 | Q2QMH1.1 | 4-262 | *Oryza sativa* |
| OsNEK3 | Q6ZEZ5.1 | 4-262 | *Oryza sativa* |
| OsNEK4 | Q60DG4.1 | 8-266 | *Oryza sativa* |
| OsNEK5 | Q94CU5.1 | 8-266 | *Oryza sativa* |
| OsNEK6 | Q6YY75.2 | 4-260 | *Oryza sativa* |
| TbNRKA | AAX79136.1 | 20-283 | *Trypanosoma brucei* |
| TbNRKB | Q03428.2 | 20-283 | *Trypanosoma brucei* |
| TbNRKC | AAY90075.1 | 4-262 | *Trypanosoma brucei* |
| TbNEK1 | XP_843901.1 | 14-273 | *Trypanosoma brucei* |
| TbNEK7 | XP_843912.1 | 20-282 | *Trypanosoma brucei* |
| TbNEK9 | XP_844954.1 | 29-298 | *Trypanosoma brucei* |
| TbNEK10 | XP_845461.1 | 53-320 | *Trypanosoma brucei* |
| TbNEK11 | XP_845981.1 | 4-264 | *Trypanosoma brucei* |
| TbNEk12.1 | XP_847541.1 | 41-303 | *Trypanosoma brucei* |
| TbNEK12.2 | XP_844679.1 | 41-303 | *Trypanosoma brucei* |
| TbNEK14 | XP_847000.1 | 6-259 | *Trypanosoma brucei* |
| TbNEK15 | XP_803439.1 | 14-303 | *Trypanosoma brucei* |
| TbNEK16 | XP_827922.1 | 4-259 | *Trypanosoma brucei* |
| TbNEK17 | XP_822838.1 | 33-303 | *Trypanosoma brucei* |
| TbNEK19 | XP_822396.1 | 33-375 | *Trypanosoma brucei* |
| TbNEK20 | XP_829176.1 | 12-270 | *Trypanosoma brucei* |
| TbNEK21 | XP_829559.1 | 78-389 | *Trypanosoma brucei* |
| TbNEK22 | XP_951524.1 | 16-288 | *Trypanosoma brucei* |
| XlNEK9 | Q7ZZC8.1 | 34-294 | *Xenopus laevis* |
