# Supplementary material for: A NIMA-Related Kinase Suppresses the Flagellar Instability Associated with the Loss of Multiple Axonemal Structures
Source: PLoS Genet. 2015 Sep 8;11(9):e1005508. doi: 10.1371/journal.pgen.1005508 (PMC4562644; doi:10.1371/journal.pgen.1005508)
Supplement: S4 Table — (DOCX) [file pgen.1005508.s006.docx]

**S4 Table: Antibodies used in this study**

| **Protein** | **Size** | **Dilution** | **Species** | **Reference** |
| --- | --- | --- | --- | --- |
| DRC1 | 79 kD | 1:10,000 | rabbit | [10] |
| DRC2 | 65 kD | 1:100 | rabbit | [10] |
| DRC3 | 60 kD | 1:1,000 | rabbit | [10] |
| DRC4 | 55 kD | 1:1,000 | rabbit | [10] |
| DRC5 | 43 kD | 1:500 | rabbit | [10] |
| DRC7 | 177 kD | 1:10,000 | rabbit | [10] |
| DRC11 | 95 kD | 1:1000 | rabbit | [10] |
| Tektin | 58 kD | 1:10,000 | rabbit | [45] |
| Rib72 | 70 kD | 1:10,000 | rabbit | [54] |
| Rib43a | 43 kD | 1:5,000 | rabbit | [55] |
| DII1/p28 | 28 kD | 1:2,000 | rabbit | [25] |
| DHC11 | >250 kD | 1:1,000 | rabbit | [46] |
| DIC3/IC140 | 140 kD | 1:20,000 | rabbit | [47] |
| RSP16 | 40 kD | 1:20,000 | rabbit | [51] |
| DLE2/Centrin | 20 kD | 1:2,000 | mouse | [50] |
| DIC2/IC69 | 69 kD | 1:10,000 | mouse | [53] |
| CCDC39 | 101 kD | 1:1,000 | rabbit | [10] |
| polyE | 50 kD | 1:2,000 (immunoblot);  1:500 (immunofluorescence) | rabbit | [28] |
| LF5 | 66 kD | 1:2,000 (immunoblot); 1:200 (immunofluorescence) | rabbit | [56] |
| α-Tubulin | 50 kD | 1:2,000 | mouse | [19] |
| Acetylated α-tubulin | 50 kD | 1:250 (immunofluorescence) | mouse | [19] |
